# Risk of Mercury Ingestion from Canned Fish in Poland

**DOI:** 10.3390/molecules25245884

**Published:** 2020-12-12

**Authors:** Aleksandra Pawlaczyk, Anna Przerywacz, Magdalena Gajek, Malgorzata Iwona Szynkowska-Jozwik

**Affiliations:** Faculty of Chemistry, Institute of General and Ecological Chemistry, Lodz University of Technology, Zeromskiego 116, 90-924 Lodz, Poland; 204307@edu.p.lodz.pl (A.P.); magdalena.gajek@edu.p.lodz.pl (M.G.); malgorzata.szynkowska@p.lodz.pl (M.I.S.-J.)

**Keywords:** canned fish, seafood, mercury, CVAAS, acceptable dose

## Abstract

In this study, total mercury content was determined in 84 canned fish corresponding to commonly consumed brands (over 14 different producers), which were purchased from local markets in Poland in the years 2019–2020. For comparison purposes, samples of both the matrix in which the fish were kept along with the seafood samples were measured. The analyses were carried out using the cold vapor AAS technique. Statistical analyses were employed to identify significant differences in mercury content in relation to the selected criteria such as fish species, type of fish (predatory, non-predatory) and the producer brand. The obtained results were compared against domestic and international standards as well as with the literature data in order to evaluate the safety of the canned fish consumption. The study revealed that none of canned fish exceeded the acceptable levels set by the FAO/WHO. The highest amount of Hg was recorded for canned tuna (maximum 351.30 µg/kg, mean 74.38 µg/kg). Further, the estimated tolerable dose of weekly mercury intake suggests that the consumption of over 1.8 cans of fish with the highest mean mercury content should not pose a risk to consumers in Poland according to international standards. Among the ten highest mean results for mercury, five of them belonged to canned tuna (Bonito species) kept in different matrices. These consisted of seven domestic and three imported brands of fish products, which is a worrying message for a local community. Mercury content in predatory fish differed significantly from the results gathered for non-predatory fish and the total amount of mercury in studied canned fish corresponded to their status in the aquatic food chain. Moreover, significant differences were stated between various fish species and fishing areas. Fish caught in the Atlantic Ocean (cod and herring) presented higher mercury content than the ones from closed seas.

## 1. Introduction

Mercury occurs in the environment in various forms: elemental, inorganic, and organic. They are all toxic but differ in their level of toxicity. The degree of toxicity depends mainly on how the form is absorbed and how it is biotransformed to other mercury forms [1,2,3]. The routes of exposure include: inhalation, ingestion (e.g., by food or water), or absorption through skin. In addition to the type of mercury being absorbed, the route and duration of exposure, the dose and the age or the developmental stage of the person exposed play a crucial role in the observed health effects [4].

There are two main sources of exposure to organic forms: through consumption of fish and other seafood products and by contact with products containing mercury compounds inhibiting bacterial growth like in cosmetics (thiomersal) [1,5,6]. It is a well-known fact that organic compounds will be more effectively absorbed from the gastrointestinal tract than inorganic forms, because of their better lipid-solubility and binding to sulfhydryl groups. When aryl and long-chain alkyl compounds are absorbed in tissues, they are transformed into divalent cations representing the toxicity of inorganic forms, whereas the most dangerous short chain alkyl mercurial compounds are absorbed in 90–95% in the gastrointestinal tract and stay stable in almost unchanged forms [1,7]. From the point of view of non-occupational mercury exposure, the primary compounds that are commonly examined are short-chained alkyl groups, such methylmercury. Public awareness about the toxicity of this compound was raised just after the first official mercury poisoning cases in Minamata Bay were documented. Exposure to methylmercury occurs almost exclusively through the consumption of fish, especially predatory fish, seafood, and meat of large marine mammals. Methylmercury accounts for approximately 70–90% of total mercury content in them according to different literature data [8,9,10] and it undergoes bioaccumulation, bioconcentration, and biomagnification along the food chain [11,12]. However, this compound can be absorbed as well through the skin and lungs. More notably, even if the inorganic forms are incorporated into the water, bacteria can initiate changes of the transformation from mercury to methylmercury, a far more toxic form [11]. The biological half-life of methylmercury is assessed to be about 65 days. The data suggest that chronic mercury poisoning for a 70 kg adult can be caused by the daily intake of methylmercury of about 0.3 mg, which may lead to increased mercury concentration reaching 0.2 mg/L in blood and 60 mg/kg in hair [1]. Inorganic compounds can be absorbed in about 10% from the gastrointestinal tract, whereas the absorption of methylmercury via gastrointestinal tract can be about nine times higher. After absorption, methylmercury is located between brain, liver, and kidney. Both organic and inorganic mercury can be excreted mainly through feces [1,3,7].

Despite the fact that fish and other seafood products can contain dangerous compounds, they are an inseparable element of the human diet. Both fish and seafood provide many wholesome and easily digestible ingredients, such as: proteins; A, D and E and B vitamins; or other necessary micronutrients, such as magnesium, phosphorus, potassium, and sodium. They are an excellent source of unsaturated fatty acids from the omega-3 group, such as EPA and DHA [2]. It seems to be vital for the people to properly balance the risk of MeHg exposure versus the benefits associated with the seafood consumption. Consumers should be aware and regularly updated regarding the potential danger. In Europe, the average consumption of fish and other seafood products is around 25.1 kg per capita per year. According to the data from 2017, the highest annual consumption of fish and seafood is in Portugal (56.9 kg/person). Similarly, a high annual consumption per capita is in Spain (45.6 kg) and in France (33.7 kg). In Poland, an average of 15 kg of fish products per capita pre year is consumed. This result is almost two times lower than the European standards. The lowest consumption is in Hungary, where the value is 5.6 kg/person/year. The most frequently consumed species of fish in the European Union per capita per year are as follows: tuna (mainly canned tuna)—3.07 kg, cod—2.31 kg, and salmon—2.24 kg (it is postulated that a significant amount of salmon originates from the fish farm). In the case of seafood, Europeans prefer mussels—1.28 kg, which are mostly farmed, and wild squid—0.67 kg [13].

The increasing awareness of the potential negative effect of mercury compounds on people’s health from food consumption led to the introduction of the so-called Tolerable Weekly Intake (TWI) by the Joint FAO/WHO Expert Committee on Food Additives (JECFA) [14,15,16,17]. The limit for an inorganic mercury of 4.0 μg/kg b.w./week expressed as mercury was established, while for methylmercury the advisable level was set at 1.6 μg/kg b.w./week. The choice of the given value was based on the epidemiological studies investigating the joint effect of maternal exposure to mercury and impaired neurodevelopment in their children [18]. The recommendations regarding the limit value assessed for methylmercury vary among the European countries and depend upon the amount and the type of fish consumed regionally. Also, the estimated intakes of methylmercury differ with the organizations, which set the limits for public, e.g., the value provided by (U.S.) National Research Council (NRC) is about two times lower than the limit given by the JECFA (0.7 µg/kg body weight per week). PTWI value respected in Poland is in agreement with the dose recommended by the JECFA [14,15,16,17]. Obviously, this dose can be exceeded even a few times for populations consuming considerable amounts of mercury and thus will differ significantly both across various age groups and nationalities. Since even for the low methylmercury levels the toxicity of this compound has been demonstrated, reliable data should be provided in order to protect the most vulnerable groups like women of childbearing age, unborn children and pregnant women, especially that fish and seafood products for these groups are recognized as an important element of their balanced diet and are frequently consumed [18].

In 2006, the European Commission Regulation provided information on maximum permissible concentrations of heavy metals in food products. In the case of mercury, it has been established only for fish and fishery products, which is in the range of 0.5–1.0 mg/kg of fresh weight and depends on the species consumed. In general, it can be concluded that for the non-predatory fish the limit is twice as low as much when compared to the value set for the predatory fish. A limit has also been proposed for food supplements, for which the mercury level cannot exceed 0.1 mg/kg [15]. Taking into account the mercury content of individual species of fish and seafood, experts from the FDA/EPA have developed a recommended weekly consumption depending on the type of food consumed. The main conclusions that were drawn based on these recommendations are summarized in Table S1 in the Supplementary Materials.

Due to the dangers of exposure to various forms of mercury, there are many reports in the literature regarding the biological mercury monitoring, which only supports the importance of this kind of data available worldwide to be regularly updated. A few examples of these studies performed over the years are presented in Table S2 in the Supplementary Materials and discussed further in this study [9,19,20,21,22,23,24,25,26].

The main aim of this work was to evaluate the influence of different factors on levels of total mercury in canned fish and their packaging purchased from local markets in Poland in the years 2019–2020. Since Hg bioaccumulation in fish and seafood depends on various parameters such as the fish type (predatory, non-predatory), species, size or fishing location, the studied samples were grouped and the obtained results were statistically tested according to the chosen hypothesis. Additionally, in this work mercury content in canned fish produced by different companies (including imported and domestic brands) was examined in order to evaluate the potential danger for the local community. The gathered results were compared with the current regulations and guidelines, as well as with the literature data in order to evaluate the safety of their consumption. In Poland, the consumption of canned fish is definitely less popular than in other European countries, thus the current data regarding the levels of mercury in canned products are quite limited. The presented findings aim to fulfil the gap by determining possible risk for local community. The data regarding advisable and safe portion size along with the consumption frequencies should be regularly updated since the pollution of our environment changes with time and within regions. Additionally, the tolerable dose of weekly intake of mercury was calculated, which makes it possible to determine whatever canned fish can pose a potential threat to the health of Polish consumers. Further research is also needed in order to clarify fully all aspects related to subsequent mercury bioaccessibility and its bioavailability, especially in the context of various ingredients accompanying consumed canned fish, which can affect the final mercury intake.

## 2. Results and Discussion

### 2.1. Validation of Analytical Methods

The calibration range was verified by checking the variances using the F-Snedecore test for the standards with the lowest and with the highest mercury content. The Dixon test was used to eliminate potential outliers, also among the results obtained for both certificate reference materials. The correctness of the applied analytical procedure was verified by the analysis of two different certified reference materials: CRM M-3 HerTis (MODAS-3 Herring Tissue) and CRM M-5 CodTis (MODAS-5 Cod Tissue) produced within the MODAS project execution in accordance with a few scientific institutions (certified within the scope of “MODAS” consortium by the Institute of Nuclear Chemistry and Technology, Warsaw, POLAND, and the Gdansk University of Technology, Gdansk, Poland). Their matrices were consistent with the matrices of the studied samples. Both types of fish (herring and cod) constituting the matrices of both CRMs were also analyzed within the presented research as the canned fish products. The CRMs were tested during the daily measurement cycle in two–three replicates each day depending on the number of samples analyzed. Thus, in total, 27 samples of each certified reference material were examined, with insubstantial differences in the mercury content among the measurement days. The generated results were applied to create two Shewhart individual control charts (Figures S1 and S2 in Supplementary Materials).

Both Shewhart individual control charts showed that all the results were within the ranges determined by the lower and upper control lines. Moreover, no significant trend of results for the obtained data set was observed, since the influence of systematic changes of the mean value was not present. All the points indicating daily results have random distribution.

The normality of the distribution for both CRMs was tested. Obtained *p*-value for an assumed significance level of 0.05 suggested that for CRM M-5 CodTis the data do not follow the normal distribution, while for CRM M-3 HerTis the opposite conclusion was drawn (the *p*-value was larger than the significance level) (Table 1).

For CRM M-3 HerTis, the most typical values were more largely distributed (a wider frame was achieved for 50% of the most typical values), with a much lower variance level when comparted to the results gathered for CRM M-5 CodTis. For the CRM M-5 CodTis, the recorded variance was much higher, which was a consequence of a significant distribution of both 25% of the lowest and 25% of the highest values acquired for this material (Figure S3 in Supplementary Materials).

For both certificate reference materials, the recovery values have been also calculated. The results are shown in Table 1.

The total mean values for all 27 measurements for both CRMs were within the confidence intervals of these materials specified by the manufacturer. The uncertainty of the certified values given in the certificates consisted of four contributions: standard deviation of the overall mean, standard uncertainty estimated from the long-term stability studies, standard uncertainty estimated from the homogeneity studies and standard uncertainty due to the moisture determination. Determined recoveries in both cases exceeded 99%. A two-tailed t-test was employed to compare the mean value with the certified value of mercury for CRM M-3 HerTis. In the case of the M-5 CodTis Kruskal–Wallis test was engaged to compare the median with the certified value for mercury. No statistically significant differences were stated.

The established quantification limit for the whole analytical procedure was set up to be 0.24 µg/kg.

To summarize, it can be stated that the correctness of the proposed analytical procedure (proper parameters of the samples atomization) used for the determination of mercury in canned fish and seafood products, was positively verified.

### 2.2. Total Mercury Content in All Studied Samples

**Figure 1:**
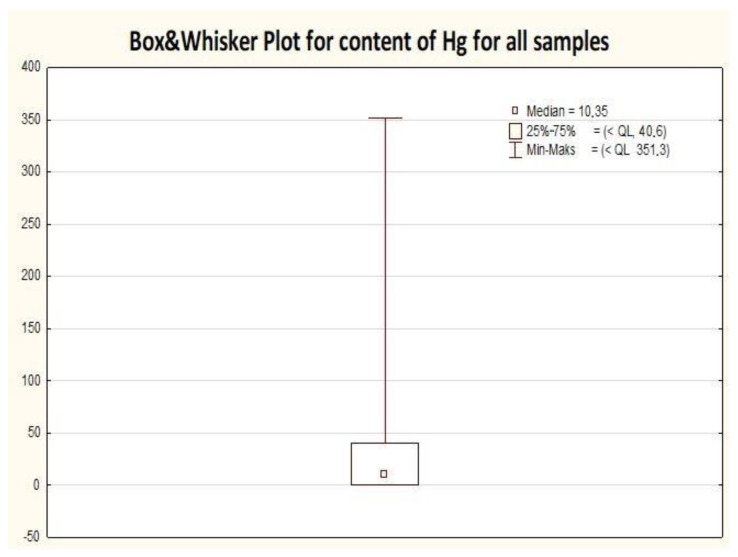
Box and whisker plot for the content of Hg for all studied samples (canned fish, matrices, and seafood products) (*n* = 489) (µg/kg).

In this study, 489 samples in total were analyzed, including canned fish products, matrix of canned fish products (e.g., jelly, oil, tomato sauce, own juice), and selected seafood products (Table 2). Each sample was analyzed in two or in three replicates. All the canned fish samples were done in triplicates. Results for the matrices were mostly done in duplicates as they did not exceed the established limit of quantification. When a good agreement was achieved for the first two replicates, the third replicate for the matrix sample was omitted. For further statistical analysis, every analytical sample was considered individually and no mean value was calculated for the primary sample (with an exception of the mean results presented in Table 3 and expressed as a mean of three independent results). All the obtained results are gathered in Table 2 and additionally presented in the form of box and whisker plots (Figure 1). For this dataset, the null hypothesis states that the data following the normal distribution can be rejected because the *p*-value was lower than the significance level of 0.05.

The distribution of all results indicated the asymmetric length of the whiskers and a visible shift of the median value towards 25% of the lowest results obtained in this study, which was confirmed by normality tests (Figure 1). The visible lack of a whisker representing 25% of the lowest mercury amounts can be justified by the significant contribution of the results below the calculated limit of quantification for the applied analytical procedure. In general, mercury was not detected in the samples belonging to the matrix of analyzed samples (such as: oils, jelly, sauces, etc.), which only supports the hypothesis that mercury does not migrate from the fish to the ambient medium. This is in agreement with the literature data. Similar findings as in our study were reported by Burger et al., 2004 [8], who also analyzed the liquid component of canned fish and obtained very low levels of mercury, usually below the MDL (method detection limit) for liquid of 0.001 mg/kg with a maximum of 0.07 mg/kg.

Moreover, the frame width for all mercury results (Figure 1) indicates a small variation of 50% of the most typical values, while the highest variation was noted for 25% of the highest results for mercury (long whisker up to the value of around 351 µg/kg). Both the length of the upper whisker and the relatively narrow box, as well as the position of the median value prompt a small quantity of results with a high mercury content.

The width distribution of the results (Table 2) can be explained by the very large value of variance when compared to the median value. In this study, the mean value was twice as high as the median and about two-fold lower than the standard deviation. The range of the results was equal to the maximum value obtained in this study, since the lowest value was below the quantification limit.

The results collected in this paper for canned fish products are comparable with literature findings, in which the increase of the Hg content with the type of meat treatment was observed. The influence of the canning process on the content of total mercury in canned tuna was investigated by Vieira et al. [25], who determined the Hg concentration in three different steps of the canning process (raw meat, cooked mean, canned). It was revealed that the content of mercury increases along the canning process in the flowing order: raw < cooked < canned). At the same time the percentage of humidity decreased along the canning process (raw > cooked > canned) no matter which kind of muscle was investigated. In white muscle the observed Hg content was 0.134 ± 0.003 μg g^−1^, against Hg content of 0.155 ± 0.003 μg g^−1^ in dark muscle. The same situation was noted by others. The significant increase in Hg concentration from raw samples to canned samples was reported by Rasmussen et al. [27], who stated the 23% increase in mercury in canned samples of albacore tuna (*Thunnus alalunga*). The average concentrations of total mercury were: 0.17 ppm (range 0.09–0.24 ppm) in the pre-canned samples and 0.21 ppm (range 0.10–0.33 ppm) in the post-canned samples. Authors postulate that the Hg concentration may be different since the humidity present in the tissues can vary. With the loss of humidity during cooking, Hg content may be increased in the meat [27,28].

It is assumed that although during culinary procedures, various constituents, such as water and fat, can be released from the seafood matrix as a result of changes in their texture and matrix (e.g., protein denaturation, Afonso et al. [29]), Hg remains strongly bonded to proteins, thus, translating into higher Hg concentrations in fish muscle (Burger et al. [28], Marmelo [29]). As it was indicated in some reports, mercury, even if it is released into the aquatic environment in an inorganic form, can be transformed into organic methylmercury via the methylation process performed by some microorganisms. Mercury reacts readily with the sulfur-containing groups (it is strongly attached to, e.g., thiol groups like in cysteine) and remains stable, and does not get destroyed by fish preparation and cooking [9]. Fish can be preserved in many ways, including freezing, salting, pickling, smoking, drying or through canning. Unfortunately, canned products may contain substantial amounts of heavy metals [19]. Canned fish are ready-to-eat products, thus notably different from fresh or frozen ones. Canned fish are subjected to a high temperature to generate the sterility. As a consequence, the obtained product is fully cooked. Fish which are supposed to be canned are delivered as fresh (salmon and herring) or frozen (tuna) products. Canned fish can be also enriched with some extra ingredients such as oils, water, or sauces, e.g., salmon are not accompanied by any additional packaging media, tuna may be packed in vegetable oil, water, or broth, while sardines and herrings can be filled with oils, flavors, or others. Proper vacuum in hermetic canned products is maintained by double-seaming machines, which generate fish integrity during heat processing, cooling and further storage at room temperature [30].

### 2.3. The Top Ten Highest Mean Results of Mercury for All Studied Samples

Deep insight into all gathered results revealed that the first ten samples with the highest mean amount of mercury (based on three replicates for each canned fish/seafood sample) belonged mostly to canned fish (nine out of ten mean mercury values) (Table 3). Not for all samples the species of fish or seafood product were indicated on the label. For example, in the case of tuna, for which the highest results were recorded in this study, the only species which was positively identified was Katsuwonus pelamis (Bonito). Canned tuna products were present in various forms: as salads, pastas, tuna in various sauces (e.g., tomato, own juice) and in oil. Only for one out of four different tuna salads investigated in this research, the tuna species was given on the can label. In all other cases (tuna in oil, tuna as pastas, tuna is various sauces), tuna species were positively identified. For this reason we compared our results with the data obtained in other related studies for Bonito species itself.

In Table 3, ten samples with the highest mercury content are listed. The codes which were applied by authors in this study are additionally explained at the end of this paper. The determined mercury content in various fish and seafood products was compared with the literature data review summarized briefly in Table S2 (Supplementary Materials).

Based on the outcomes shown in Table 3, five out of ten of the highest mean mercury content originated from the canned tuna and they were within the following range 100–336 µg/kg. It does not seem surprising since tuna is identified as a predator, able to bioaccumulate in the tissues considerable amounts of heavy metals, including mercury and, thus, representing a significant dietary source of this element to humans. Tuna, due to its position in the food chain, and as a result of their swimming habits with their mouths open can absorb mercury more effectively throughout their life [9]. The obtained mean mercury contents in this study are more or less comparable with the results reported by others, where the bonito species was investigated. For instance, Rodriguez-Mendivil et al. [31] determined H_TOT_ in canned tuna. The highest value which was achieved in the mentioned work reached 278 µg/kg, which was similar to the highest mean result reported in this work. Also, results obtained in our study (Table 3) for other investigated canned fish were similar to those described for the same fish types elsewhere (Table S2 in Supplementary Materials). According to Suppin et al. [32], the highest mercury content that was determined in herring was about 150 µg/kg, while for cod the maximum mercury concentration reported was 40 µg/kg. Surprisingly, in the group presenting the ten highest mercury levels also the results gathered for pollock were included, even though it is not a predatory fish. Mean mercury content in this case was about 127 µg/kg. Other literature reports indicate that, for this fish, the mercury content can be much lower (10 µg/kg) [33]. The possible deviations can result from the differences in the species, size and fishing location. In the list presented in Table 3, also a seafood representative can be found. The mean mercury content in octopus was about 91.5 µg/kg. A similar value was given by Lourenço et al. [34] who reported the maximum amount of mercury reaching 140 µg/kg.

As it was already mentioned, all of the five tuna samples listed in Table 3 belonged to Bonito species, but they were kept in different matrixes (tomato sauce, oil, own juice). Bonito tuna commonly refers to a light tuna, which lives for a shorter period of time, therefore, less mercury can be accumulated in its tissues. For example, Thunnus alalunga has a maximum weight of around 40 kg, which is achieved at the age of 15, while for Katsuwonus pelamis the maximum weight of about 30 kg is obtained at the same age [35]. For this reason, light tuna (which in cans can be present as a mix of different species such as yellowefin, tongol, and sometimes big-eyed) is recognized as one of the healthiest canned fish option available on the market. It is a well-known fact that larger tuna fish, like yellowfin or albacore, which are commonly identified as the so-called white tuna, can contain much higher content of mercury because for them it takes much more time to reach maturity. Thus, much larger species of fish will present much higher concentrations in their organs as the last part of the food chain in water. The average size of fish will be different among the species. For example, it is postulated that eight-year-old yellowfin (Thunnus albacares) reaches about 175 kg, whereas bigeye (Thunnus obesus) can gain approximately 210 kg at 15 years of age [36]. For this reason, we can assume that if, in this study, other species than Bonito would have been analyzed, probably much higher mercury levels would be achieved for canned tuna products.

Tuna (mostly canned) has been the most-consumed fish in the EU for the last decade. Approximately 86% of EU consumption of fresh fish and seafood is shared by 12 EU countries with the same leader in the consumption since many years—Spain. Poland takes eighth place regarding the consumption of fresh fish and seafood. When the actual consumption of fish and seafood species in the EU (expressed as the fresh weight equivalent) is considered then, apart from tuna, other fish and seafood are most eagerly eaten and they can also be placed in the following order: cod, salmon, Alaska pollock, herring, clams, mackerel, hake, squid, prawns [37]. Therefore, taking into account the amount of the worldwide annual consumption and the increase of awareness of the potential consumers about the side effects as well as some confusing and insufficient information indicated on the labels of canned products, it seems to be desirable to monitor the amount of mercury on a regular basis.

The presence of canned fish samples in Table 3 with completely different matrices may suggest that the type of packaging medium does not influence the mercury content. It should be additionally highlighted that in any of the analyzed samples mercury was detected, even for fish samples packed in its own juice. From the consumer’s point of view, fish packed in water is sometimes not preferable since the loss of Omega 3 fatty acids may occur. Moreover, water-packed fish may have less refined taste. For this reason, oil-packed fish is favorable and enables sustaining the nutrients within the fish (e.g., long-chain n-3 fatty acids or high-quality protein). It is also sometimes postulated that fish in their own juice can present a significant threat to our health, when compared to samples packed in other matrices. However, it is necessary to mention that such a conclusion cannot be found in the literature. In literature data, even if canned fish in various types of fish matrices are examined, they are often not distinguished, hence, any comparisons with other findings are quite difficult [20,38]. However, there are some limited data to which we can refer, which suggest no influence of the packaging medium on the mercury content in canned fish [9,21,26]. This issue was extended in this paper in the section dedicated to the results obtained only for the canned tuna.

Among the ten highest mean results for mercury, the indicated products listed in Table 3 consisted of seven domestic and three imported brands, which is a worrying message for the local community. Additionally, two out of seven domestic brands are local, while five of them have their production in Poland, but they are the representatives of other international companies in our country. The remaining three brands offer the canned fish produced in Germany (pollock), UK (tuna), and Thailand (tuna). Moreover, five species included in Table 3, like Bonito, Atlantic cod, Atlantic herring, Atlantic Pollock, and Octopus vulgaris, seem to be imported by most Polish producers. Two brands appeared in this list twice with two different and one common product (tuna, cod, and herring). It can be suggested that in Poland various brands import fish products from the same source since the products bought from the local market, but produced by different brands and packed in different media have similar mercury content. For example, tuna salad produced by brand coded as “OO” exhibited comparable mercury content (156.6 µg/kg) to the same tuna species (Bonito) manufactured by different Polish company and named as brand “ZZ” (129.4 µg/kg). This finding supports the hypothesis that mercury content in our study is mainly affected by the species, size, and fishing area. It is worth noting that for any of the ten mean highest results (Table 3), the maximum permissible concentration of mercury in food products was exceeded. For mercury, the established levels are in the range of 0.5–1.0 mg/kg of fresh weight depending on the fish type [15]. According to some reports, the practical admissible level of mercury in food products can be decreased, while only products with a considerable amount of mercury have to be labeled [9,39]. Shim et al. [39] postulated that the maximum level of mercury content for the commercial fish should be decreased to 0.185 mg/kg. In addition, these authors suggested that due to the susceptibility of young children to the toxic effects of mercury, products with low mercury concentration (such as mackerel) should be specially marked as “kidsafe”. According to González-Estecha et al. [21] it is safer for children and pregnant women to consume canned mackerel, which has a much lower mercury content than tuna. In our study, the results obtained for canned mackerel were within the range of 1.7–90.5 with a mean of 40.9 (µg/kg), which are considerably lower than those obtained for canned tuna. The lowest result obtained for mackerel was reported for a salad, while the highest ones were recorded for sauce and oil matrices (no pastes mackerel pastes were collected in this work).

What should be also emphasized is the fact that the current recommendations given in the regulation refers to raw products, which may lead to wrong estimation of the risk of dietary exposure. However, in the future approach it is crucial to consider the bioaccessibility and bioavailability data, which can help to correctly establish the official recommendations. In this case, factors like various culinary treatments and a possible interaction with different ingredients, that make a final meal should be taken into account. Canned fish are products ready to be eaten, so by delivering more current data that can be used in a realistic risk assessment of canned fish products, consumers are enable to make more conscious dietary decisions. Thus, in some countries, due to a very high level of mercury in fish products and the possible health threat connected with mercury poisoning, studies aiming at mercury determination are performed continuously or intermittently. In one work, studies concerning the determination of mercury and methylmercury in canned tuna produced by various brands of the Persian Gulf were conducted and the results were compared to the data gathered for the same canned tuna brands in previous years. It was proven that generally a decrease in the mercury content was observed for five brands and for two an increase in mercury content was indicated [9].

### 2.4. Results Collected for Canned Tuna, the Influence of the Packaging Medium

**Figure 2:**
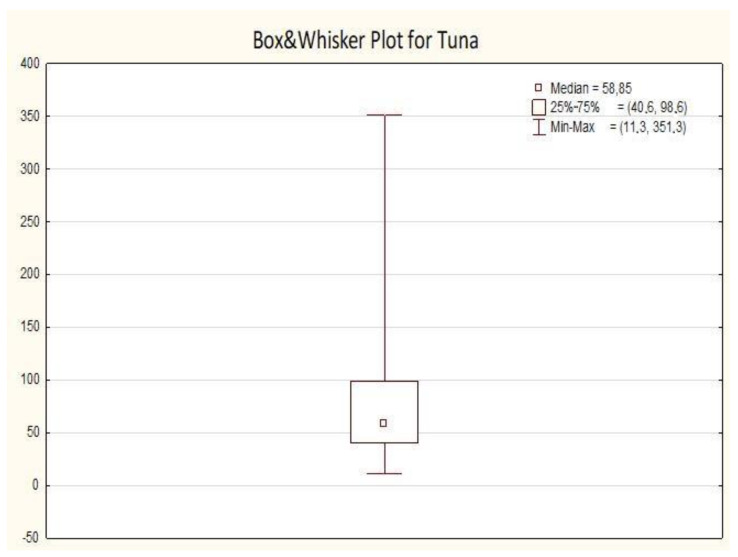
Box and whisker plot for content of Hg for canned tuna produced tuna produced in different countries, including Poland (*n* = 57) (µg/kg).

All the results collected in this study only for canned tuna are presented below on the Figure 2 and Table 4.

The greatest diversity for the results was noted for 25% of the highest results (the results were in the following range from 98.6 to 351.3 µg/kg). About 75% of the results contained mercury at a level far below 100 (µg/kg), which should be a satisfactory result from the consumer’s point of view. It is necessary to mention that even much higher values of mercury in canned tuna fish were reported in other related studies, as presented, but also various species of tuna were investigated there, not only Bonito [40]. Some studies did not distinguish between different tuna species, or type of muscle, hence the comparison with the literature data is difficult in some cases [40]. The maximum value which was achieved in our study is for the canned tuna from the Bonito species. It is higher than typically recorded for this fish. However, the mean and median values obtained in our study for canned tuna (Bonito) are in agreement with the literature data. Without doubt, the majority of results were within the values proposed by U.S. Food and Drug Administration (FDA). According to them Hg levels in canned tuna fish are normally in the range 0.1–0.2 [µg/g] [8,40] and are similar to those summarized in Table S2 in the Supplementary Materials. Unfortunately, as it was mentioned by Hellberg et al. [40], studies on canned tuna do not typically allow researchers to know the size of the fish or the catch location, which can be key factors related to mercury in fish products. Also, in the study by the González-Estecha et al. [21], mercury concentrations in the “light tuna” were actually much higher than those reported by other authors (overall median 0.298 mg/kg). González-Estecha et al. [21] noted that before making recommendations regarding the consumption of fish for vulnerable populations, the concentration of mercury in the varieties of canned tuna most frequently consumed in Spain should be considered.

In the case of tuna, the lowest mercury contents were indicated in canned tuna pastes, which seem to pose less threat from the exposure to mercury perspective. Even though the mean values of mercury content in canned tuna seem to be comparable with other reports, due to a serious danger of mercury poisoning among some vulnerable groups, like children and pregnant woman, continuous monitoring, especially for these groups and among people declaring high fish consumption, should be undertaken enabling a direct comparison of the current results with some other periodic studies.

It should be also mentioned that samples of canned tuna investigated in this work were produced by six different countries, including Poland, and based on the collected data some variation of the results was observed. Moreover, five of the highest mean results obtained for canned tuna belonged to three countries: Poland, UK, and Thailand. Since tuna in our country is an imported fish (not domestic), the highest results obtained for this fish produced by Polish brand is quite disturbing. This diversity of mercury contents in the canned tuna samples from various localities requires a long-term monitoring program to provide region-specific information on contaminant levels to the public to enable them to make decisions about which fish to eat to avoid the health risk as it was suggested by Kumar [41].

In our study, the only species of canned tuna which was positively identified was Bonito (Katsuwonus pelamis). This tuna is the smallest among other tuna species and extremely popular in terms of fishing since bonito represents 58% of the tuna catches in the world. It reproduces very quickly, reaching maternity at the age of 1–2 years. Bonito is caught mostly in the tropical waters of the Pacific, Atlantic, and Indian Oceans. Due to its small size this species is most often used in the production of canned food [42]. The importance of the fishing location with regard to the mercury content in tuna fish was shown in a few papers. For example, Kumar [41] postulated that oceanic tuna species (albacore, bigeye, bluefin, skipjack, and yellowfin) are more commonly investigated than coastal tuna species (kawakawa, blackfin, little tunny, frigate tuna, and longtail tuna). Significant disparity probably resulted from greater commercial importance of oceanic tuna species. Based on the literature review dedicated to the total mercury content that was presented by this author, it can be concluded that coastal marine environment is highly contaminated by heavy metals, including mercury, leading to high metal accumulation in coastal species when compared to oceanic ones [41]. It should be noted that since 13 December 2014, the rules for labels accompanying all fishery and aquaculture products for EU consumers have changed. In terms of processed fishery products like canned fish, the information regarding fishing area displayed in the bar codes is not mandatory but voluntary, in contrast to the, e.g., unprocessed fish products, for which the catch area has to be provided [43].

In order to assess the influence of the packaging medium on mercury content, the results obtained in our study only for canned tuna samples were later grouped according to the matrix used. The samples were grouped into four classes: in oil matrix (OL), as salads (SAL), in sauce (e.g., tomato dip or own juice) (SOS) and as pastes (PAS). In our study, the highest values of mercury, the biggest mean value and the largest variation of results were stated for tuna packed in oil. Within this group, also the greatest number of samples was investigated. However, no statistically significant differences were observed for the mentioned groups, which suggests that mercury content is not affected by the packaging medium (Figure S4 in Supplementary Materials). A similar conclusion was drawn by Gerstenberg et al. [26], who verified the differences in mercury content in canned tuna within one brand, but kept in different packaging medium. Obtained concentrations of Hg [26] were compared between 10 cans of chunk white tuna in water and 10 cans of the same brand and type in oil. Statistical analysis revealed no significant difference between canned tuna packed in oil (mean of 0.807 ± 0.298 mg/kg) and canned tuna packed in water (mean concentration of 0.579 ± 0.330 mg/kg) due to a large amount of variance within the two groups, although the mean concentrations were different. Burger et al. [8] compared the results of many brands of tuna, which were packed in water and oil. For none of the comparisons statistically significant differences were indicated, even though the authors observed that the average content of mercury in oil-packed tuna was lower for white and higher for light species. Some authors also suggested that fresh tuna or tuna steaks can contain higher concentration of mercury than canned tuna. With respect to the packaging medium, González-Estecha et al. [21] did not identify any statistically significant differences in mercury medians among samples packed in olive oil, sunflower seed oil or in the water packaging medium, as well as among mercury medians in olive oil, sunflower seed oil, and the water packaging medium. Dezfouli et al. [9] tested 45 canned tuna fish of 10 different companies. In his work, five, three, and two brands of studied tuna cans packed with soy, olive, or vegetable oil, were studied, respectively. However, no influence of the packaging medium on the mercury content was found, similar to our study.

### 2.5. Results Collected for All Canned Fish, the Influence of the Brand and the Country of Production

In this study, all the canned fish and seafood products belonged to 25 different brands (six for the seafood products and sushi, and 19 for canned fish). Both types of analysis were performed: with and without the seafood products. In both cases, no statistically significant differences between the mercury content and brand type were recorded. In the final stage of the brand influence analysis, all results only for canned fish were selected. The dataset was grouped in such a way that brands represented by only one canned fish were classified into the same group (“others”). As a consequence, 13 different brands of canned fish (each brand was represented by more than one canned fish product) and one “other brand” (to this group all six various brands were assigned, for which only one canned fish was collected) were identified in this study. Again, there was no difference among the mercury contents in canned fish of different brands. This finding is in good agreement with the previous studies reporting no significant influence of brand factor on mercury content [9]. However, some authors have suggested that the possible variation of the results within different brands of canned fish may be attributed to the age of fish. According to Okyere et al. [19], who determined mercury content in 180 samples of canned fish comprising 28 brands of mackerel, 25 brands of sardines, two brands of pilchards, and five brands of tuna purchased in Ghana, the analysis of variance showed a significant difference in mercury concentration within the species represented by particular brands. Authors concluded that mercury levels were affected by the fact that some canned fish were older than others. Low levels of mercury corresponded to products obtained from waters not yet significantly polluted with mercury. Among other parameters, the influence of brand was also investigated by numerous authors. As in the case of our study, no statistically significant differences for the mercury content determined in canned tuna products of 10 different brands were reported by Dezfouli et al. [9]. Moreover, the same authors revealed that factors, such as the expiration date, packaging environment, price of the product and the production season, did not influence the mercury content. A similar conclusion was drown by other authors. In the study by the González-Estecha et al. [21], 36 cans of tuna of the most popular brands in Spain were analyzed. The influence of the type of tuna, its forms (olive oil, sunflower seed oil, water, or marinade), brands, prices, and expiration dates were investigated. However, they found no differences between packaging medium, brands, prices, or expiration dates. Opposite outcomes were revealed in another report by Gerstenberger et al. [26], who determined the mercury content in 155 cans of tuna from three national brands: brand 1 (*n* = 54), brand 2 (*n* = 46), and brand 3 (*n* = 55) purchased in Las Vegas, USA. The statistical test indicated that brand had significantly higher Hg concentrations (mean of 0.777 ± 0.320 mg/kg), while the Hg concentrations of brand 1 (mean of 0.541 ± 0.114 mg/kg) and brand 2 (mean of 0.550 ± 0.199 mg/kg) were statistically similar.

All the results gathered for canned fish products were then grouped according to the country of production (Figure S5 in Supplementary Materials). The majority of samples collected in this study originated from Poland. Analysis of variance showed significant differences in mercury content between samples produced in MA and PH, MA and PL, MA and TH, MA and GER and MA and UK. For samples produced in Morocco, the smallest variation of results was observed. This could be attributed to the fact that for this group only one type of fish was investigated, which belonged to the non-predatory group (sardine made by three different companies). The highest results which were obtained for countries, such as Thailand (TH), Germany (GER), Philippines (PH), United Kingdom (UK), and Poland (PL), were gathered only for predatory fish. For samples produced in TH (three brands), PH (two brands), UK (one brand) only canned tuna was purchased. The results for GER (one brand) concerned only one type of canned fish (pollock), while for PL (11 brands) seven types (cod, herring, sardine, salmon, tuna, mackerel, sprat). The highest variation of results was received for canned fish produced in Poland. This is the only country for which seven out of eight investigated types of fish were collected, namely cod, herring, sardine, salmon, tuna, mackerel, sprat. The influence of the location of fishing on the mercury content was already raised in this paper in the section dedicated to the analysis of mercury content in canned tuna.

**Figure 3:**
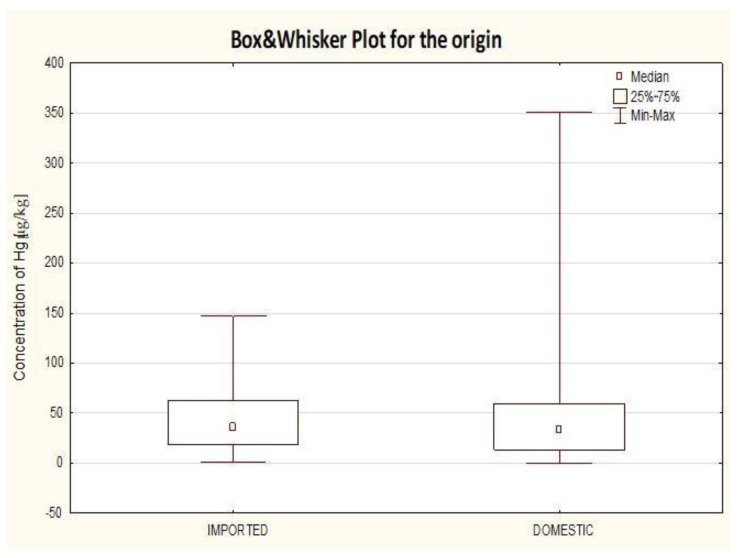
Box and whisker plot for content of Hg for canned fish produced in Poland (domestic, *n* = 192, median = 36.6) and outside Poland (imported, *n* = 60, median = 33.4; total *n* = 252) (µg/kg).

Subsequently, the results gathered for canned fish were divided into two groups: domestic (canned fish produced in Poland) and imported (canned fish with the production outside Poland) (Figure 3). It needs to be stressed that this division is not an equivalent of the division into species which is typical of Poland, e.g., caught from the Baltic Sea and the ones for which the fishing area in not located around Poland. That kind of classification was not possible to be applied due to the lack of information regarding the fishing area and species in all cases. Imported canned fish consisted of the following fish species: pollock, tuna, salmon, sprat, sardine and mackerel. No statistically significant differences were stated between mercury content and the sample origin (imported vs. domestic). The highest diversity of the results was recorded for 25% of the highest results gathered for domestic production (much higher diversity) and for 25% of the highest results obtained for imported brands. In both cases (local and imported), the highest results belonged to canned tuna. Much smaller variation of the results for imported products stems from the number of samples analyzed and much greater diversity of studied fish types when compared to the data collected for local brands.

### 2.6. Results Collected for All Canned Fish, the Influence of the Type of Fish (Predatory vs. Non-Predatory)

**Figure 4:**
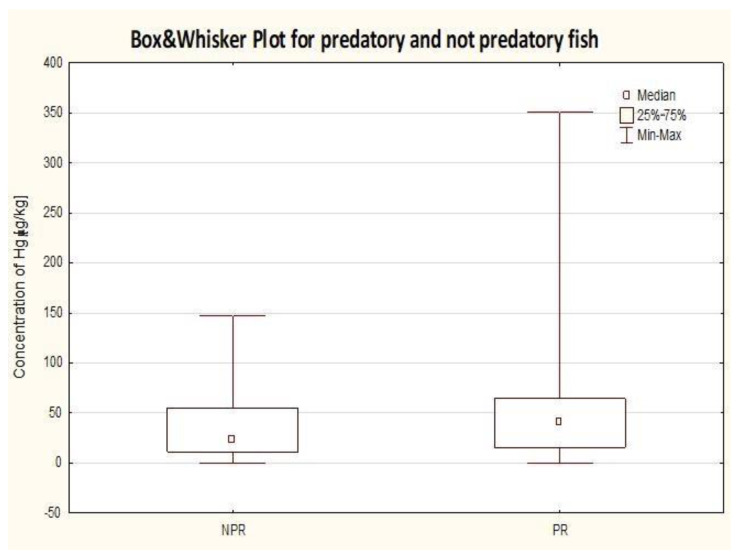
Box and whisker plot for content of Hg for predatory and non-predatory fish (NPR—non-predatory fish, *n* = 105, median = 24.1; PR—predatory fish, *n* = 147, median = 44.7; total *n* = 252) (µg/kg).

In the next step of our analysis, all the results gathered for fish samples were divided into two separate groups according to the type of food consumed: predatory (PR) and non-predatory (NPR) (Figure 4). In the predatory group, the following fish were included: tuna, salmon, cod, and mackerel, while in the non-predatory group: sprat, herring, sardine, and pollock. Related studies evidently show that large predatory fish, which are on the top of the food chain, can pose a substantial risk to human being as a significant source of mercury exposure.

Not surprisingly, in our study for the predatory species the highest individual results of mercury concentration were recorded. For this group, the median value was directed towards the highest values of mercury concentration, while the biggest statistical dispersion was noticed within 25% of the highest results. A similar tendency regarding the distribution of data was stated for the non-predatory group; however, in this case the median value was clearly headed toward the lowest values measured. For predatory fish, the median value was almost as twice high as that for the non-predatory group and the preformed analysis of variance showed significant differences in mercury concentrations between both groups. High value of variance, in the predatory group in particular, can be attributed to the fact that within this group fish species with different size, diet, and level of food consumption were represented. Obviously, in reality, many species of the fish consume mixed food no matter if they are predatory or not. Numerous observations of fish nutrition prove that almost all non-predatory fish, with very few exceptions, under certain circumstances eat other fish eagerly, juvenile fish in particular, even those of their own kind. On the other hand, typical predators sometimes consume invertebrates as well. Nevertheless, the mercury content in any case did not exceed the acceptable international standards, thus, it can be concluded that consumed canned products available on the Polish market do not pose a significant public health concern.

In the next step, the dispersion of the results within both studied groups (predatory and non-predatory) was studied individually. For the non-predatory fish, the highest values of mercury were recorded for pollock fish, a member of a cod family (Figure S6 in Supplementary Materials). This semi-pelagic schooling fish lives typically in the cold northern waters of Pacific seas even at depths up to 1300 m. This fish lives no longer than 15 years, weighs about 1.5 kg, and reaches approximately 40–45 cm, but some adults from the Bering Sea can reach as much as 75 cm. The rest of the studied fish species from the non-predatory group belongs to the herring family (sprat, herring, and sardine). Sardine typically lives in the Mediterranean, Marmara, and Black Sea, as well as in the waters of the Pacific and Indian Oceans. This fish is quite small, with an average length of just 15–20 cm, weighing up to 50 g. Sardines are supposed to live up to ten years but the abundant year classes peak at about four or five years of age. They can migrate along the coast but live near the coastal area. Sprat is a sea fish also from the herring family. It lives in the eastern part of the Atlantic Ocean, as well as in the Northern, Baltic, and Northern Mediterranean and Black Seas. The size of sprat is 10–20 cm and its weight is about 15 g. Life expectancy does not exceed eight years. Herring, the last representative of the herring family, commonly occurs in northern areas (Atlantic and also in the Baltic Sea). The body length of herring is 15–30 cm and its weight is about 400 g. It lives up to 30 years and scavenges to a depth of 250 m [35].

In our study the size of the fish was one of the most important factors affecting the results since the mercury content increased in the group along with the size of the fish, as follows: pollock (mean 127.33 µg/kg) > herring (mean 47.8 µg/kg) > sprat (mean 16.1 µg/kg) > sardine (mean 11.4 µg/kg). Among the listed fish in our study, the largest one is pollock, even though it lives twice as long as herring. The possible diversity of the obtained mercury levels in this fish may be a consequence of the low number of analyzed samples. The highest diversity of the results for 50% of most typical values was also recorded for the herring fish, which was connected with the fact that samples of various species were analyzed only for this type of fish with a completely different natural place of living (Baltic and Atlantic herring). Analysis of variance proved the existence of statistically significant differences in the concentration of mercury between herring and sprat and between pollock and sprat. For pollock and herring, the highest variances were observed, while for sprat the lowest variation for this group was reported.

In addition to the fish size, the diet or level of fish consumption by various species, other potential reasons for higher mercury levels should also be taken into account. It should be kept in mind that both potential migration of some species and the place of residence can have a direct impact on the differences in mercury levels. For instance, bottom-dwelling fish may contain more mercury due to the proximity of the bottom sediments, in which the mercury is deposited. Literature data regarding mercury mobility do not provide clear answers; however, it is speculated that mercury accumulated in sediments possibly under favorable conditions can be released into solution [44,45]. Thus, contaminated bottom sediments constitute a potential source of contamination under specified conditions. Moreover, the location of fishing can affect the mercury level since the mercury concentration in fish will be correlated with the mercury levels of the surroundings. The degree of anthropogenic and natural activities leading to the release of mercury and the water flow can significantly influence the mercury content in fish. Due to the fact that locations of fishing are not known to the public, and in many cases are not indicated on the labels, the potential comparisons are quite difficult at this stage of analysis.

The same type of analysis was then made for predatory fish (Figure S7 in Supplementary Materials), and as it was already mentioned all the samples recorded mercury concentrations below the accepted limit recommended by the FAO/WHO and adopted by Poland. In this group four types of fish were investigated: tuna, mackerel, cod, and salmon. Tuna belongs to the mackerel family and the typical place of residence of this fish are the Atlantic, Pacific and Indian Oceans, as well as the Mediterranean Sea, definitely being less common in the cooler waters of the North or Barents Sea. The medium length of tuna is about 2–2.5 m, and the weight is about 350 kg. Tuna can live up to 30 years, but due to intensive fishing not so many achieve a given limit, which also affects the risk of their extinction. What is worth mentioning from the mercury level point of view is the fact that tuna is a migratory fish (search for food). Mackerel is also the member of the mackerel family, which typically lives in the Atlantic Ocean or Mediterranean, Black, and Baltic Seas. This fish is much smaller when compared with tuna. The size of mackerel is usually up to 50 cm and its weight is about 1 kg. The average lifespan of this fish does not exceed five years. These fish are a migratory species that can cover a distance of up to 500 km. Moreover, they can live at depths of up to 1000 m below the sea level, close to the bottom, where they look for the food. Cod is the largest predatory sea fish in the cod family. Its natural environment is the Atlantic Ocean, but it can also be found in the North Seas. The average body length is about 30–80 cm, and the length can reach 2 m, with the average weight of this fish being about 0.8–2 kg (maximum up to 40 kg). Cod can live as long as 20 years and, most importantly, this fish feeds on the bottom, which can have a direct impact on mercury content in their muscles. Cod is a typical bottom fish among this group of four different predatory fish analyzed in this study. Salmon belongs to the salmon family and live in the northern parts of the Atlantic Ocean, North, White, and Baltic seas, and it can be found in some rivers, like in North America. It reaches 150 cm, while their weight fluctuates within the limit of 24 kg. Salmon live up to about 13 years and are famous for their wandering, which significantly affects mercury content in their bodies. Intensive farming of Atlantic salmon began over 50 years ago in Norway [35]. Cultures developed rapidly, first in Europe and then outside Europe, like in Chile and Canada. After a significant market saturation, nowadays it is estimated that aquaculture, or farming, accounts for as much as two-thirds of total salmon production. Moreover, according to the current data of the European Commission, as much as 68% of the fish and seafood we consume comes from outside the European Union, and only 10% is fish and seafood from farms on the continent. The question which is raised at this point is the type and quality of food used to feed the farmed salmon vs. wild ones and what kind of impact it may have on the content of some pollutants.

The highest levels of mercury among all predatory fish were determined in the canned tuna and the obtained data seem to be not alarming when we compare the data with the results collected by other authors. Relatively good agreement was achieved with data presented by Dezfouli et al. [9], who reported the highest concentration of about 315.2 µg/kg for canned tuna fish purchased in Iran. Okyere et al. [19] showed that the highest value of mercury in canned tuna also obtained from local markets in Iran was about 470 µg/kg. Significantly higher values were indicated by Chen et al., 2011, [46] with the highest values exceeding 3 mg/kg in muscles of Thunnus obesus for samples purchased in Taiwan. However, these authors investigated various species of tuna, with no indication of the size of fish or type of muscles studied. One of the highest values which was reported in the literature for canned Skipjack species from Hong Kong reached 469 µg/kg with a range of results within 0.037–0.469 mg/kg and mean value about 0.163 mg/kg [47].

Thus, the highest result obtained for canned tuna in our study, when only Bonito species is considered, can be a little bit worrying but not alarming, as it was already stated, since this value is still below the recommended limits. In our study for this fish (canned tuna), the largest variation of results was noted. It can be a consequence of a different size and age of studied fish. Moreover, in the case of this fish not for all samples the information regarding species was indicated for tuna salads, so we cannot exclude that the mix of species was used instead of one species. Surprisingly, in our work quite a wide diversity of the results for total mercury content was also stated for cod fish. It is quite possible that cod fish with different size and age and also belonging to different species were analyzed. The cod species were positively identified by us (Black and Atlantic cod) and the observed differences were shown later in this work. Based on our study, it was very difficult to correlate in all cases the fish size and the mercury content. The mean mercury content for studied fish was as follows: tuna > (mean 79.7 µg/kg) > cod (mean 53.9 µg/kg) > mackerel (mean 40.9 µg/kg) > salmon (mean 12.6 µg/kg), which did not correspond with the fish size. In this group, also other relevant parameters should be included, like the migration of the fish or the type of fish (e.g., bottom or coastal).

For this group of four predatory fish, analysis of variance was made in order to verify potential differences in mercury concentration among studied fish. Performed analyses showed that there are statistically significant differences among the results gathered for salmon and tuna, salmon and mackerel, and salmon and cod. Within the salmon group, the smallest variation of results was reported (very narrow box for 50% of most typical values). We cannot exclude that this fish can originate from the same location of fishing, and even though it was produced by five different brands, the cod fish could have been from the same source.

### 2.7. Results Collected for All Canned Fish, the Influence of the Fish Species

Among all investigated fish samples, only for two representatives (herring and cod) were we able to positively identify more than one species. Thus, in the next step of data analysis only these canned fish were included, for which the species was indicated on the label (the rest of the results were then rejected). For herring, classified as a non-predatory fish, we have distinguished two species: Baltic (HERB) and Atlantic (HERA). Although only nine individual samples of Baltic herring were analyzed (when compared with 33 samples of Atlantic herring), the existence of statistically significant differences was stated (Figure S8 in Supplementary Materials). The variance of the results of mercury content in Atlantic herring was over seven times higher than for the Baltic herring. The mean mercury content for Atlantic herring was about three and a half times higher (mean 61.7 µg/kg) than for Baltic herring (mean 16.9 µg/kg), which is optimistic information for the local fish market in Poland. It should be kept in mind that other parameters, such as dietary composition of fish along with the global mercury emissions can affect Hg levels even for the same fish species. Other authors also indicated that there might be some differences in mercury regarding their place of residence. For instance, Chen et al. [46] compared the results of mercury in tuna fish from the Atlantic Ocean with the same species but from the Indian Ocean. Both total mercury and organic mercury levels for samples originating from the Atlantic Ocean (mean Hg_TOT_ = 898 µg/kg fresh weight) were significantly higher than those from the Indian Ocean (mean Hg_TOT_ = 679 µg/kg fresh weight).

For the predatory fish, we were able to distinguish more than one species only for cod fish, namely Atlantic and Black cod (Figure S9 in Supplementary Materials). The same tendency was recorded for herring, even though a much smaller number of samples was analyzed in this case. The analysis of variance revealed the existence of statistically significant differences between the results gathered for Atlantic and Black cod. Atlantic species again was characterized by much higher median and variance (mean 82.4 µg/kg) when compared with Black species (mean 8.20 µg/kg). Based on the above-mentioned results for herring and cod fish, it can be reported that mercury content varies with the species of the fish due to the different location of fishing and, in both cases, Atlantic species were characterized by higher mercury content. This can be quite surprising since it is assumed that coastal species will have higher mercury content than oceanic ones [41], as is discussed later in this paper.

In total, all canned fish were purchased from more than 14 different brands (as it already explained in details in this paper). Not for all brands we were able to collect all information regarding all investigated types of fish. For instance, in the case of cod fish two different species were analyzed, which were at the same time produced by two different companies. Thus, no relation was found between mercury content and a brand since the observed differences were determined mostly by the species of the cod fish. We decided to check the potential influence of the producer on mercury content for the fish for which a bigger number of samples collected like for the herring. For this fish, six different brands were identified, and the statistically significant differences were stated for the following pairs: between brand MM and EE, between brand ZZ and EE, between brand FF and EE. All the indicated differences were again the consequence of the herring species since brand EE and NN offers only Baltic herring, while the other companies sell Atlantic herring (Figure S10 in Supplementary Materials). It again supports the hypothesis about the lack of influence of the brand on the mercury content, being mostly limited by the fish species characteristic of the specific fishing location.

### 2.8. Results Collected for Other Seafood Products

For comparison purposes, some seafood products were also investigated (Figure S11 in Supplementary Materials). The highest results regarding mean, median, and maximum values were obtained for octopus samples, while the lowest for mussels. The largest variation of the results within both 50% most typical values and 25% of the highest mercury results was also stated for octopus. Only for this type of seafood, the median value was not directed towards the 25% of the lowest values. Additionally, quite high mercury content was detected in crabs. Within this group, the lowest variance in mercury content was noticed, which is in agreement with the literature data. According to Kot et al. [48], high concentrations of mercury and methyl mercury were also reported in seafood, especially in octopus and crabs.

In many reports, the data considering other seafood products than fish are given. The study of the Baltic mussels Mytilus trossulus living in the Bay of Puck was undertaken in 2019 by Jedruch et al. [49]. Mussels tissues were freeze-dried and then homogenized. The obtained concentration of total mercury Hg_TOT_ was 44.9–670.6 ng/g, while the median value reached 144.5 ng/g. These results are almost in line with other studies conducted for the same species of mussels in the Bay of Puck. A relationship was also found between the Hg_TOT_ concentration in mussels tissues and the size of their shell. Smaller organisms were characterized by not only much lower mercury concentrations, but also by better quality and, consequently, higher nutritional value. In our study the results gathered for mussel samples were much lower and did not exceed the value of 35.0 µg/kg. However, when this kind of result is compared to the published data, special attention should be paid to the sample preparation, which can dramatically influence the mercury content. 

### 2.9. Methylmercury Contribution into the Total Mercury Content in Fish Based on the Literature Data—Bioaccessibility, Absorption, and Metabolism

In this study, only total mercury content was determined in canned fish and other seafood products. Obviously, high concentration of mercury in fish can be a result of its bioaccumulation (net accumulation of a trace element in a tissue of interest or a whole organism that results from exposure), bioconcentration (uptake of a chemical by an organism directly from the abiotic environment resulting in a higher concentration within the organism), and the position of fish in the food chain (biomagnification being an increase in the concentration in an organism from a lower trophic level to a higher trophic level within the same food web due to bioaccumulation from the diet), as it was previously mentioned [11,12]. It is also a well-known fact that methylmercury can be a dominant form of mercury in fish. Some authors suggest that this organic form may account for over 90% of the total mercury and by many this amount of mercury is wrongly treated as equal to the bioaccessible fraction. It was shown that the levels of mercury in fish are the highest in well-perfused tissues like liver, spleen, and kidney [50,51], but the highest concentration of mercury in their organisms seem to be in muscle. The literature data indicate that, in fish muscle tissue, over 95% of the Hg(II) is in the form of MeHg [52], where mercury is mostly bound to the thiol group of the cysteine residues in fish proteins [53] and present as MeHgCys (MeHg-cysteine complexes) [54]. Thus, this particular form of mercury seems to be the most crucial for humans as it comes from the consumption of fish and seafood products and it is assumed that processes like cleaning or cooking the fish do not eliminate strongly-bonded mercury. For instance, Dezfouli et al. [9] determined the mean mercury and methylmercury contents in 10 different canned tuna brands. The assessed methyl mercury to total mercury content ratio reached from 77.8% to 84.7%. However, no information was given in this study about the degree of the bioaccessible fraction. The presented results were generally in agreement with other literature data. Salaramoli et al. [55] indicated that methylmercury constitutes about 80% of total mercury in tuna fish. In the work of Storelli et al. [10], the value of 91% was given (which accounted for an average amount of methylmercury), similar to the data obtained by Burger et al. [8] in canned tuna with 89% (average) achieved. Marmeloa et al. [29] determined the total mercury content in complete meals of tuna against in sewed tuna alone as well as the bioaccessible Hg in the same meals. They applied in vitro digestion model described previously by Alves et al. [56]. In their work, each sample was digested in three steps, including: (1) Oral phase, where saliva fluid was added to the fish; (2) gastric phase, where gastric fluid was added; and (3) intestinal phase, where duodenal fluid and bile fluid were added. The authors determined the bioaccessibility percentage after the in vitro digestion, which amounted from 13.5% for stewed tuna up to 42.2% for raw tuna.

The slow process of elimination of MeHg by fish, described in a few papers [35,36,57] and the efficiency of MeHg transfer from food to fish may both be responsible for higher levels of mercury determined in older and larger fish [52]. In the paper by Bradley et al. [38] it was strongly stated that even though the organic form of mercury is absorbed from the intestine definitely more efficiently than Hg(II), it does not mean that the whole amount of mercury will be absorbed from the diet. There are some factors which should be taken into account when the dietary assimilation efficiencies (AE), defined as the difference between the absorption process being a result of an amount of a chemical retained by fish after dietary exposure of a known quantity of food and the elimination process, is analyzed. The most probably type of food composing the source of mercury exposure may have a direct influence on AE. It should be underlined as well that three main processes play a key role in the mercury transfer along the food chain namely: bioaccessibility, absorption, and metabolism. Bioaccessibility is this fraction of consumed mercury which was ingested and released in the gastrointestinal tract from the food matrix into a soluble form. Absorption can be treated as a transfer of the bioaccessible compound into and across the intestinal epithelium into blood, while metabolism which may take place during digestion or solubilization as well absorption can significantly change the final contribution of the bioavailable fraction. To summarize, we can say that MeHg remains the most dominant form of mercury in fish muscle and its bioaccessibility can be applied as a quite general estimation of the bioavailability, since bioaccessibility is recognized in theory as a maximum possible bioavailability.

### 2.10. Estimation of the Acceptable Dose

As it was already discussed in the introduction part of this study, the so-called Tolerable Weekly Intake (TWI) by the Joint FAO/WHO Expert Committee on Food Additives (JECFA) [14,15,16,17] was introduced that the amount of mercury delivered from the fish and seafood products can be independently verified and compared with adopted dose intake and further recommendations. The limit for inorganic mercury of 4.0 μg/kg b.w./week expressed as mercury was established, while for methylmercury the advisable level was set at 1.6 μg/kg b.w./week. These values are the doses widely accepted in Poland [14]. Based on the literature data, about 90% of the Hg existing in predatory marine fish is in the form of methylmercury [25,50]. According to Joint FAO/WHO Expert Committee on Food Additives, JECFA, 95% of this amount of methylmercury, which is present in predatory marine fish, is rapidly absorbed and assimilated through the gastrointestinal tract and easily penetrated into blood-brain and placental barriers in humans and animals. Therefore, it can be assumed that if considerable amounts of fish containing high levels of mercury are consumed, some undesirable human health effects may occur [25]. There is no agreement regarding the level of methylmercury in fish and other seafood products. Not in all studies was the total mercury content determined along with methylmercury, as is shown in Table 5. In some papers an assumption is made to calculate the contribution of methylmercury to the total mercury content. Burger et al., 2004 [8] measured the methylmercury content only in a subset of tuna cans (purchased from a New Jersey grocery store) and found that methylmercury composed 83–89% of the total mercury. As a consequence, the authors decided to apply the correction factor of 1.12 instead of analyzing methylmercury in all samples. Since their work was not primarily designed to evaluate organic mercury, the methylmercury content was finally roughly estimated by dividing the determined total mercury in canned tuna by 1.12 [8]. To show the diversity of the results regarding the methylmercury contribution, only a few examples are given since this issue was discussed by us in the previous paragraph. According to Dezfouli et al. [9], methylmercury content in canned tuna accounted for an average of 80.6% (range from 77.8 to 84.7%). Different related studies proposed various methylmercury content in tuna products when compared to total mercury content. Salaramoli et al., 2012 [55] proposed that methylmercury reached 80% in canned tuna samples collected of the Persian Gulf. Storelli et al. [10] reported the average level of methylmercury in tuna from the Mediterranean Sea of 91% with a wide range from 75% to 100%. Due to the large discrepancy of the results and to simplify the calculations in order to assess the possible level of methylmercury in studied samples, the total mercury content for canned fish and seafood products was divided by a factor of 1.12, as suggested by Burger et al. [8].

In our study, the following formula was used to evaluate the exposure to methylmercury through fish and seafood consumption [25,58]:

$$\frac{\mathrm{Amount}\;\mathrm{of}\;\mathrm{fish}\;\mathrm{ingested}\;\mathrm{per}\;\mathrm{week}\;\left(\frac{\mathrm{kg}}{\mathrm{week}}\right)*\;\mathrm{Mercury}\;\mathrm{concentration}\;\mathrm{in}\;\mathrm{the}\;\mathrm{fish}\;\mathrm{ingested}\;\left(µg/\mathrm{kg}\right)\;}{\mathrm{Kilogram}\;\mathrm{body}\;\mathrm{weight}\;\left(\mathrm{kg}\;b.w.\right)}=\;\mathrm{MeHgintake}$$

Obviously, the exposure to mercury will be a result of: (1) The amount of ingested fish per unit time (day or week); (2) the Hg concentration in the fish; and (3) the body weight of the fish consumers [25,58]. As recommended by Kuras et al. [14], the person weighing 70 kg (b.w.) was included in the calculations. In the study of Vieira et al. [25] a body weight of 60 kg based on USEPA guidelines was used.

Based on the data contained in the standards, a tolerable weekly intake of mercury (TWI) was calculated for a person with an average weight of 70 kg related to the consumption of products with the highest Hg content. When determining the %PTWI, the consumption of no more than one can of fish was assumed. To again simplify the calculations the average mass of can of about 200 g was assumed. The results are summarized in Table 4. Due to the fact that the main form of mercury in fish and seafood products is the organic form, our results referred to the tolerable weekly intake established for methylmercury of 1.6 μg/kg b.w./week.

According to the formula given by the Vieira et al. [25], the maximum values of fish meals per week without exceeding the established RfD by international agencies can also be calculated using the following formula:

$$\frac{\mathrm{Reference}\;\mathrm{dose}\;\mathrm{RfD}\;\mathrm{expressed}\;\mathrm{in}\;\frac{\mu g}{\mathrm{kg}}\mathrm{per}\;\mathrm{day}*\;b.w.\;\left[\mathrm{kg}\right]*7\;\mathrm{days}\;}{(\mathrm{Canned}\;\mathrm{meal}\;\mathrm{size}\;\left[g\right]*\left(MeHg\;in\;fish\;expressed\;in\;µg/\mathrm{kg}\right):1000}=\;\mathrm{Meals}\;\mathrm{per}\;\mathrm{week}$$

Taking into account the highest mean Hg concentration obtained in this study for the canned tuna in oil, it can be stated that calculated real MeHg intake for a 70 kg person corresponds to 53.63% of the limit established by JECFA (MeHg exposure of 1.6 µg MeHg kg b.w.^−1^ week^−1^). Considering the maximum amount of canned tuna (number of meals) for the 70 kg potential consumer per week without exceeding the RfD established by JECFA, we can conclude that it is possible to eat over 1.8 cans of canned tuna per week (200 g each can) without exceeding the RfD of JECFA. Although, the assumed average body mass of a typical consumer was higher (70 kg) than suggested in some reports (60 kg) [25], and the adopted average can mass of 200 g (due to much lower content of fish, sometimes reaching only about 60%, while the rest is packaging medium) the recommended dose of mercury in studied products was still not exceeded. The obtained results in this study were quite similar in terms of the advised safe number of meals or MeHg intake to the literature reports (Table S2). For example, Okyere et al. calculated the PTWI also for the highest concentration obtained in this study (nampa tuna fish 0.2 mg/kg wet weight). It was revealed that for an adult who weighs 60 kg about 1500 g of the nampa tuna (150 g per can) should be consumed (which is around 10 cans) to exceed 300 µg of mercury a week. In our work, for a 60 kg adult and a 150 g can the recalculated real MeHg intake would reach 46.7% of PTWI, which gives, at the same time, a maximum number of meals of 2.13. Those values calculated for both scenarios are quite comparable and do not change the conclusions that were drawn. In the study by Kuras et al. [14], the gathered results for the average consumers were also within the PTWI of methylmercury (MeHg) and, according to the authors, the risk of adverse effects form the fish consumption is considerably low. In this work, the calculated mean weekly intake (EWI) was estimated at 0.62 μg/kg b.w./week (range of 0.36–0.96 μg/kg body weight (b.w.)/week) after the consumption of four edible marine fish species every day (for 10 days) by the participants (*n* = 67) living in Lodz, Poland. The Hg intake amounted to 38.6% of the provisional tolerable weekly intake (PTWI) (1.6 μg/kg b.w., weekly) value. It is worth mentioning that participants in this study ate during the ten days approximately 1.6 kg of fried fish (a mix of four marine fish species, such as Patagonian, pollock, cod, coalfish), which came in a form of frozen filet samples from the Polish market and were caught from the Pacific Ocean. In any case the permitted maximum concentration of Hg in the studied fish was not exceeded, even after the cooking process. Moreover, the average increase in the whole blood (Hg-B microgram per liter) of the volunteers from 0.62 to 1.28 μg/L was observed. The authors also reported the increase in the mean concentration of Hg in hair (Hg-H microgram per gram) of the volunteers between the first day of fish consumption to be (0.24 μg/g), and one month after the end of the study (0.29 μg/g). As it was indicated in the work by Kuras et al. [14], the biological levels of mercury in blood and in hair will be increased due to higher fish consumption.

In the case of the dose evaluation, the data for the most vulnerable groups should be presented as well. In the WHO report [18], some data regarding the consumption for the whole population understood as eater and non-eater groups (including general population, children ≥ 6 years old and women of child-bearing age) and for the eaters only (including general population, children ≥ 6 years old and women of child-bearing age) expressed as g/kg b.w./day) were provided by the USA and New Zealand. In Table 6 below, the values which were used by us to calculate the dose for these mentioned groups are presented. The dietary exposure to methylmercury in this report was calculated for specific fish species, for which the occurrence data and consumption data were available. The data quoted by us refer only to the canned tuna. We used the values of the average consumption for both eaters + non-eaters and eaters only and estimated our own weekly dietary exposure for investigated populations. For this purpose, the highest mean MeHg content for canned tuna in oil (300 (µg/kg)) was used as a reference value. It was revealed that the calculated dietary exposures for the whole population (eaters + non-eaters) were less than the PTWI of 1.6 μg/kg b.w. for the general population, children, and women of childbearing age both for data delivered by the WHO and for data reported in our study. Assessed dietary exposures for “eaters only” exceeded the PTWI in all cases for WHO data, and only in the group of children consumers, for data obtained in this work.

Summarizing the results of the analysis, it can be assumed that the tested products do not pose a hazard with respect to the Hg content, assuming that the canned fish and seafood products are consumed in a reasonable way. Although the results presented in this work demonstrate that canned fish samples which are consumed in Poland are safe according to the European Commission limits for mercury, increasing global pollution and misleading information on the labels of products justify the need for continuous monitoring of this element in fish products. We should it keep in mind that for us, being at the end of the food chain, the final mercury concentration may, with time, increase to hazardous levels, especially when species with high mercury are consumed frequently (Agah et al., 2007) [59]. 

## 3. Materials and Methods

### 3.1. Samples

Eighty-four canned fish corresponding to 25 commonly consumed different brands and producers were purchased from local markets in (Lodz, Poland) in the years 2019–2020. For comparison purposes, samples of both the matrix in which the fish were kept along with the seafood samples were measured. All canned fish were measured in triplicate. Results for the matrices were mostly done in duplicate as they did not exceed the established limit of quantification. Therefore, there was no need to perform the third analysis. In the case of pastes, no data were generated since it was not possible to separate the matrix. In total, 489 individual samples were obtained. For further statistical analysis every analytical sample was considered individually and no mean value was calculated. Only the top highest results for mercury obtained in this study were presented as the mean values.

The whole set of samples consisted of the seafood and sushi products (by six different brands), and the canned fish products (19 brands). Most of the statistical analyses were performed for the group of canned fish. In order to evaluate how the brand may affect the mercury content, the dataset was grouped in such a way that if the brand was represented by only one canned fish it was automatically classified into the same group (“others”). As a consequence, 13 different brands of canned fish (each brand was represented by more than one canned fish product) and one “other brand” (to this group all six various brands were assigned, for which only one canned fish was collected) were distinguished in this study.

The choice of goods was dictated by their popularity, availability and price. Each sample was described in details (Table 7) so that in the further statistical analysis such factors as follows were taken into account: the fish species (separate analysis was done for both species of cod and herring), brand type (analysis was made for the top highest mean results and for canned fish separately; also, to simplify the analysis, the division was made between domestic and imported brands), country of production (details were presented for the top ten mean mercury content and for all canned fish), type of packaging medium (the influence of the matrix was shown for the canned tuna samples), and the type of fish (canned fish were divided into predatory and non-predatory).

### 3.2. Samples Preparation and Equipment

#### Mercury Analyzer

In this study, an MA-3000 automatic mercury analyzer (Nippon Instruments Corporation, Tokyo, Japan) was used to determine total mercury content in fish and other seafood samples. This instrument does not require any wet-pretreatment of the sample. In the MA-3000 the gold amalgamation method is used to capture atomic mercury after the thermal decomposition of the sample matrix. After the mercury is adsorbed by gold, the trap is heated to release the mercury and then swept by the flow of pure oxygen, being the carrier gas, into the optical cells of the detector, where the mercury concentration is measured by atomic absorption spectrometry (AAS). The basis for determination of the mercury concentration by AAS is the resonance absorption of the Hg-atoms at a wavelength of 253.7 nm. In the applied system, dual-cell and tri-detector optics are used. There are two separate calibration curves for each range, one for each absorbance cell. The linear range is split between the long cell (for lower amounts of mercury up to 10 ng) and the short cell (for the mercury content exceeding 10 ng). There are two separate calibration curves for each range. The results are always generated for both cells and the appropriate range is then selected based on the amount of mercury present in the samples, which can be modified as well by the sample mass. In the MA-3000 instrument the three detectors (long cell detector, short cell detector, and reference detector) are applied. One reference detector is to further stabilize the background from the light source, and then an independent detector is dedicated for each of the absorption cells (with short and long optical paths). In this work, all the measurements were made for the lowest rage up to 10 ng of mercury. Before measurements, the instrument was warmed up for several minutes so that the appropriate temperatures of individual furnaces were set. The calibration curve was made based on the standard solution of mercury with a nominal concentration of mercury of 0.1 mg/kg (Wako Pure Chemicals Industries, Takeda, Osaka, Japan) in the matrix of the L-cysteine solution (Nacalai Tesque, Kioto, Japan) used as a stabilizer. The calibration curve consisted of standards containing 0 ng, 1 ng, 2 ng, 5 ng, and 10 ng of mercury (specified volumes of standards containing mercury were introduced into the ceramic boats after the proper dilution of the base solution of mercury). The atomization parameters were selected and adjusted for the type of sample being analyzed (different for canned fish and seafood products and for water-based standards). All the ceramic boats used as sample carriers were cleaned with a brush allowing the removal of residues from the thermal decomposition of the samples and then rinsed with deionized water with a small amount of concentrated nitric acid (by beaker). They were then subjected to a high temperature of 680 °C for a minimum of 1.5 h to remove traces of mercury from the previous analyses. The hot boats were placed in a desiccator, where they remained until the analysis. The correctness of the conducted procedure was examined by analyzing two certified reference materials: CRM M-3 HerTis (MODAS-3 Herring Tissue) and CRM M-5 CodTis (MODAS-5 Cod Tissue) and a good agreement was achieved between certified values and obtained results. The CRMs were analyzed during the daily measurement cycle in two or three replicates each day depending on the amount of samples being analyzed. Each individual sample was taken from different locations of the canned product. The measurement was conducted the same day the canned fish were opened. Each sample was drained using a towel paper and stainless steel fork. Then three analytical samples of fish meat and from two to three samples of packaging medium—e.g., tomato sauce or vegetable oil—were distinguished. All the studied samples were weighed on the analytical balance. The weight of each sample was between 0.05 and 0.10 g.

### 3.3. Data Analysis

STATISTICA 12 (Stat Soft, Inc. Tulsa, OK, USA) software was used for statistical analysis. Normality of distribution was tested using Kolmogorov–Smirnov and Shapiro–Wilk tests. A *p*-value less than 0.05 was considered statistically significant. Due to the lack of a normal distribution for the results of mercury content in fish and seafood, the Kruskal–Wallis non-parametric test was used to verify the potential differences in mercury content among studied groups in relation to various parameters such as: fish species, fish type, packaging medium, country of production, or brand.

All the gathered data classified according to various criteria were visually presented in the form of the box and whisker plots. This type of diagram shows quite well the observed differences within the created groups in relation to the chosen parameter. The box represents results within the range of 25–75% results, whilst the whiskers show 25% of the lowest and 25% of the highest results, respectively. The greater variation of 50% of the most typical values is reflected by the greater width of the box itself. The longer the mustaches are, the higher dispersion of 25% of the lowest/highest results obtained in this work.

## 4. Conclusions

None of the samples tested in this study exceeded the permitted mercury concentration in fish and other seafood products. The highest result which was obtained (mean out of three independent replicates of 336 µg/kg) belonged to tuna canned product packed in the vegetable oil. The Bonito species is commonly recognized as the safe option for potential and frequent consumers. In the top ten mean highest results, six were reported for the predatory fish) in different packaging medium (five for tuna and one for cod fish). No mercury was detected in the matrices studied, which suggests that mercury is strongly bonded to the proteins in the canned fish products and no transfer occurs into the adjacent environment. Statistically significant differences were revealed with regard to the type of fish (predatory and non-predatory) or the species analyzed (for cod and herring). Fish caught in the Atlantic Ocean presented higher mercury content than the ones from closed seas. Neither the country of production nor the brand affected the mercury content. It should be also noted that for none of the studied samples, the maximum permissible concentration of mercury in the food products was exceeded. The tolerable weekly intake of mercury for an individual weighing about 70 kg for none of the samples analyzed was exceeded. Considering the highest result obtained in this study for the canned tuna in oil, exceeding the tolerable weekly intake would result in eating more than 1.8 cans within seven days. The estimated tolerable dose of weekly mercury intake suggests that the consumption of canned fish in Poland should not pose a risk according to the international standards.

## Figures and Tables

**Table 1:** Summary of the main statistics for the results of mercury content in both certificate reference materials (*n* = 27) (µg/kg).

CRM	*n*	Mean	Median	Min	Max	Stand. Dev.	Range	*p*-Value	Certified Value +/− Uncertainty	Recovery (%)
M-3 HerTis (µg/kg)	27	227	225	216	238	5.0	22.4	0.93	227 ± 21	99.9
M-5 CodTis (µg/kg)	27	309	309	290	329	8.4	38.9	0.03	310 ± 22	99.6

**Table 2:** Basic statistics for mercury results obtained for all tested samples (*n* = 489) (µg/kg).

*n*	Mean	Median	Min	Max	Stand. Dev.	Range	Variance
489	25.9	10.4	<QL	35	40.1	351	1607

**Table 3:** A summary of the highest mean values of mercury content in the studied samples. Values are expressed as a mean +/− standard deviation of the three independent analyses (*n* = 3) (TUN-tuna, POL-pollock, CD-cod, HER-herring, OCT-octopus, PR-predatory, NPR-non predatory, MOL-mollusc, POL-Poland, GER-Germany, UK-United Kingdom, TH-Thailand) (µg/kg).

No.	Fish Code	Species	Type Code	Matrix	Brand Code	Country Code	Hg Content (µg/kg)
1.	TUN	Bonito	PR	Oil	HH	POL	336
2.	TUN	Bonito	PR	Salad	OO	POL	156
3.	TUN	Bonito	PR	Paste	ZZ	POL	129
4.	POL	Atlantic	NPR	Fish sticks	GG	GER	127
5.	CD	Atlantic	PR	Fish sticks	OO	POL	118
6.	TUN	Bonito	PR	Oil	TT	UK	107
7.	TUN	Bonito	PR	Own juice	MM	TH	100
8.	HER	Atlantic	NPR	Sauce	FF	POL	100
9.	OCT	Vulgaris	MOL	Mollusk	AA	POL	91.5
10.	HER	Atlantic	NPR	Salad	ZZ	POL	86.4

**Table 4:** Basic statistics for all mercury results obtained for canned tuna produced in different countries, including Poland (*n* = 57) (µg/kg).

*n*	Mean	Median	Min	Max	Range	Variance	Stand. Dev.
57	79.7	58.8	11.3	351	340	5174	71.9

**Table 5:** Summary of TWI values for products with the highest mean mercury content (TUN-tuna, POL-pollock, CD-cod, HER-herring, OCT-octopus).

No.	Fish Code	Mean Total Hg Content (µg/kg)	Mean MeHg Content (µg/kg)	Intake of Total Hg per Can 200 g (μg)	Tolerable Weekly Intake (TWI) (μg/kg b.w./week.)	Real MeHg Intake for a 70 kg Person (mg/Person/Week)	% PTWI	Meals per Week
1.	TUN	336	300	67.3	1.60	858	53.6	1.88
2.	TUN	156	139	31.3	1.60	399	24.9	4.00
3.	TUN	129	115	25.8	1.60	330	20.6	4.85
4.	POL	127	113	25.5	1.60	324	20.3	4.93
5.	CD	118	105	23.6	1.60	301	18.9	5.30
6.	TUN	107	95.5	21.4	1.60	272	17.1	5.86
7.	TUN	100	89.8	20.1	1.60	256	16.0	6.24
8.	HER	100	89.4	20.0	1.60	255	15.9	6.27
9.	OCT	91.5	81.7	18.3	1.60	233	14.5	6.85
10.	HER	86.4	77.1	17.3	1.60	220	13.8	7.26

**Table 6:** The estimated weekly dietary exposures to methylmercury for the whole populations (eaters and non-eaters) and for eaters only (μg/kg b.w./week) (PTWI: 1.6 μg/kg b.w./week). The data are calculated in relation to the canned tuna of Skipjack species. The mean content given in the WHO report for Skipjack canned tuna was 0.14 mg/kg, while the maximum value reached 0.49 mg/kg.

Canned tuna	Consumption—Average (g/kg b.w./Day) for Whole Populations	Weekly Dietary Exposure for Whole Populations (μg/kg b.w./Week) (Average Concentration × Average Consumption × 7)	Weekly Dietary Exposure for the Whole Populations (μg/kg b.w./Week) (Max Concentration × Average Consumption × 7)	Weekly Dietary Exposure for the Whole Populations in Our Study (μg/kg b.w./Week) (Max Concentration of MeHg × Average Consumption × 7)	Consumption—Average (g/kg b.w./Day) for Eaters Only	Weekly Dietary Exposure for Eaters Only (μg/kg b.w./Week) (Average Concentration × Average Consumption × 7)	Weekly Dietary Exposure for the Eaters Only (μg/kg b.w./Week) (Max Concentration × Average Consumption × 7)	Weekly Dietary Exposure for Eaters Only in Our Study (μg/kg b.w./Week) (Max Concentration of MeHg × Average Consumption × 7)
General	0.04	0.04	0.12	0.08	0.57	0.56	2.0	1.2
Children	0.04	0.04	0.13	0.08	1.1	1.1	3.8	2.31
Women of CB	0.03	0.03	0.10	0.06	0.49	0.48	1.7	1.03

**Table 7:** Characteristics of tested samples in relation to their origin.

Type				
Fish (252 Individual Samples)		Seafood (105 Individual Samples)		Matrix (132 Individual Samples)
Predatory (147 Samples)	Non-predatory (105 Samples)	Molluscs (75 Samples)	Shellfish (30 Samples)	
Salmon (Atlantic)	Pollock (Alaska)	Squid (Indian)	Prawns (Kidi)	Jelly
Tuna (Bonito)	Sardine (European)	Mussel (Chilean)	Crab sticks	Oil
Mackerel (Atlantic)	Sprat	Octopus (Vulgaris)	Crab sticks (smoked from sushi)	Floods vinegar
Cod (Atlantic)	Herring (Baltic)			Sauce: -Tomato juice -Chili sauce -Cream sauce -Dill sauce -Mustard sauce -Salsa sauce -Own juice
Cod (Black)	Herring (Atlantic)			
Cod (smoked from sushi)				

## Supplementary Materials

The supplementary materials are available online.

## Abbreviations

CD	Cod
HER	Herring
TUN	Tuna
POL	Pollock
SAL	Salmon
SPR	Sprat
MAC	Mackerel
SAR	Sardine
PRA	Prawn
SQ	Squid
MUS	Mussel
PR	Predatory fish
NPR	Non-predatory fish
JE	Jelly
FV	Floods vinegear
Hg_TOT_	Total mercury
MeHg	Methylmercury
POL	Poland
GER	Germany
UK	United Kingdom
TH	Thailand
F	Fish
SF	Seafood
b.w.	Body weight
d.w.	Dry weight
f.w.	Fresh weight
MA	Macocco
EC	Ecuador
LV	Latvia
IT	Italy
NOR	Norway
