# Supplementary material for: Risk of Mercury Ingestion from Canned Fish in Poland
Source: Molecules. 2020 Dec 12;25(24):5884. doi: 10.3390/molecules25245884 (PMC7764699; doi:10.3390/molecules25245884)
Supplement: Supplementary file 1 [file molecules-25-05884-s001.pdf]

Table S1. The list of fish and seafood products classified according to the recommendations proposed by the Commission Regulation No. 1881/2006 based on the mean levels for certain contaminants in the foodstuffs, which were determined in these products.

The best choice		A good choice		Avoid	
**Product**	**mean Hg [mg/kg]**	**Product**	**mean Hg [mg/kg]**	**Product**	**mean Hg [mg/kg]**
Atlantic mackerel	0.05	Halibut	0.24	King mackerel	0.73
Cod	0.11	Carp	0.11	Marlin	0.49
Squid	0.02	Spanish mackerel	0.35	Swordfish	1.00
Crab	0.06	Tuna white (can)	0.35	Shark	0.98
Prawn	0.01	Tuna white: fresh, frozen	0.36	Tuna big-eyed	0.69
Salmon (can)	0.01				
Salmon: fresh, frozen	0.02				
Mollusc	0.01				
Pollock	0.03				
Sardine	0.01				
Herring	0.08				
Tuna light (can)	0.13				

Table S2. A brief review of the literature on the mercury content of canned fish [mg/kg].

Total mercury [mg/kg]	Sample	MeHg [mg/kg]	Additional information	PTWIs	Reference
Range: 0.079 – 0.315; mean 0.177	Canned tuna (n = 40)	Range: 0.071 – 0.268, mean: 0.144 (mean MeHg/Hg: 80.6%)	Different brands from the Persian Gulf (10 in total), Teheran, Iran	Not determined	[9]
Range: < 0.01 – 0.08 (mackerel, n = 84); range: 0.01 – 0.04 (sardine, n = 75); range: 0.01 – 0.03 (pilchards, n = 6); range: 0.12 – 0.20 (tuna, n = 15)	Canned fish (n = 180)	Not determined	Local and imported brands, 28 brands of mackerel, 25 brads of sardines, 2 brands of pilchards, 5 brands of tuna, Kumasi, Ghana	Max. for Nampa tuna (0.2 μ/g), adult 60 kg has to eat 1500 g (150 g per can, approx. 10 cans) in order to accumulate 300 μg of mercury	[19]
They assumed that the total mercury concentration found in the fish species analysed is actually methylmercury	Samples from fresh, frozen and canned fish and shellfish products (n = 485)	Median range for canned products 0.0 (clam, frigate, mussel, octopus, sardine, squid) – 0.222 (tuna)	11 species of canned products, Granada, Spain.	For a person weighing 60 kg: 63.63 μg/week	[20]
Range:<br>Light tuna: 0.205 - 0.594<br>White tuna: 0.276 - 0.558<br>Skipjack: 0.299 - 0.322<br>Fraigate tuna: 0.182 - 0.257	Canned tuna (n = 36)	Not determined	The most popular brands in Spain. Four type of tuna: light tuna, white tuna, skipjack, frigate tuna, Madrid (32) and Cartagena (4), Spain	Not determined	[21]

Range: 0.0378-0.5243 (mean: 0.2087)	Canned tuna (n = 50)	Not determined	Popular brands of canned tuna from the Morocco	For a person weighing 60 kg: 0.67µg/kg b.w./week	[22]
Albacore (mean 0.326 and range 0.174 – 0.507, n = 49) Skipjack (mean 0.132 and range 0.057 - 0.305, n = 21) Yellowfin (mean 0.066 and range 0.046 – 0..095, n = 14) Not specified (mean 0.095 and range 0.016 - 0.237, n = 72) Total (mean 0.170 and range 0.016 – 0.507, n = 156)	Canned tuna (n = 156)	Not determined	16 stores in Toronto and Vancouver (Canada)	Not determined	[23]
Range 0.02 - 0.47; mean 0.123 (domestic brands); range 0.02 - 0.11; 0.047 (imported brands)	Canned tuna (n = 54),	Not determined	Domestic and imported brands (10 in total), Teheran, Iran	Not determined	[24]
Skipjack tuna fish: Range 0.117 - 0.157 and mean 0.115 in the white muscle; range 0.133 - 0.171 and mean 0.124 in the dark muscle.	Canned tuna (n = 30)	Not determined	The comparison was made among mercury content in raw, cooked and canned fish. Also the differentiation was made between Hg content in white and dark muscles, Aveiro, Portugal	It is possible to consume 10 cans of white muscle per week (78 g each can) without exceeding the RfD of JECFA and 8 cans when considering the USEPA RfD. For the dark muscle, the number of canned tuna meals per week is lower.	[25]
Mean for Brand 1: 0.777 ± 0.320 Mean for Brand 2: 0.541 ± 0.114 Mean for Brand 3: 0.550 ± 0.199	Canned tuna (n = 150)	Not determined	Three national brands: B1 (n = 54); B2 (n = 46); B3 (n = 55). B1 chunk white (n = 10); B1 chunk light (n = 15) B3 oil (n = 10); B3 water (n = 10), Las Vegas, USA	For 25-kg child: - 75g tuna containing 0.5 mg/kg Hg per 15 days - 75g tuna containing	[26]

Mean for Brand 1 (chunk light): 0.264 ± 0.006<br>Mean for Brand 1 (chunk white): 0.502 ± 0.086;<br>Mean for Brand 3 (oil): 0.807 ± 0.298;<br>Mean for Brand 3 (water): 0.579 ± 0.329	0.777 mg/kg Hg per 23.3 days

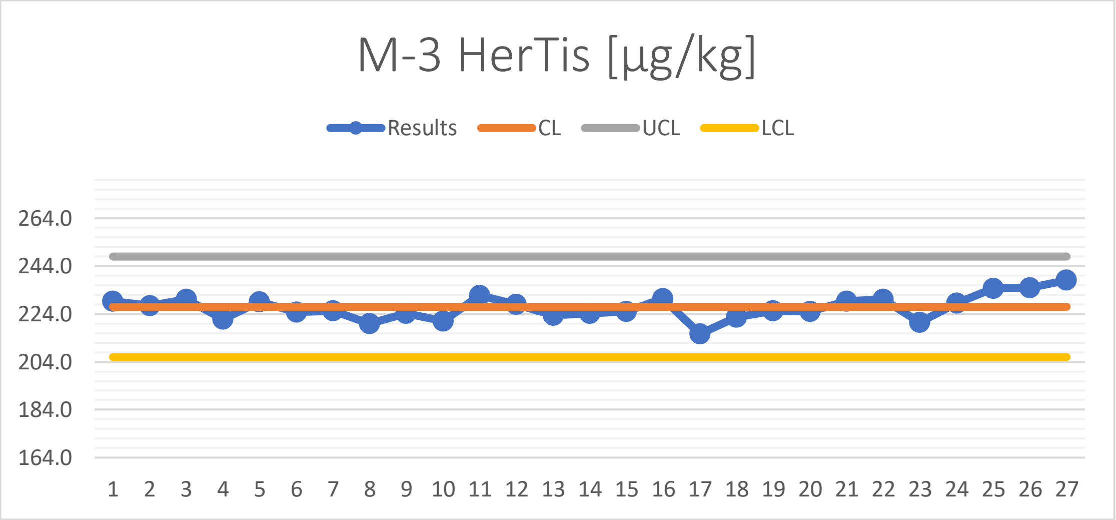

Figure S1. Shewhart limit chart for mercury content in CRM M-3 HerTis among the measurement days, where *centre line* (CL) is a certified content of mercury, *upper control limit* (UCL) is a certified content of mercury + an uncertainty, *lower control limit* (LCL) is a certified content of mercury - an uncertainty.

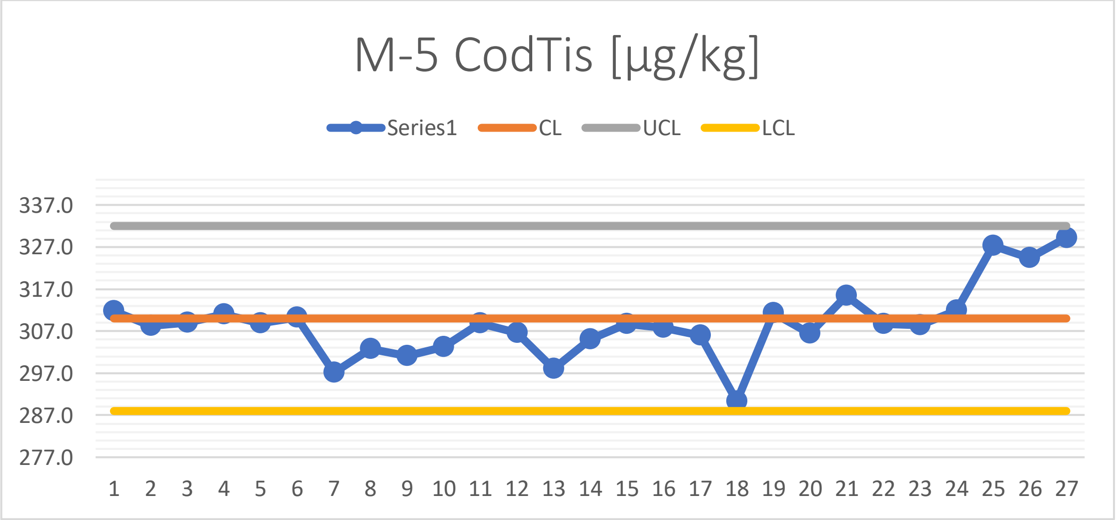

Figure S2. Shewhart limit chart for mercury content in CRM M-5 CodTis among the measurement days, where *centre line* (CL) is a certified content of mercury, *upper control limit* (UCL) is a certified content of mercury + an uncertainty, *lower control limit* (LCL) is a certified content of mercury - an uncertainty.

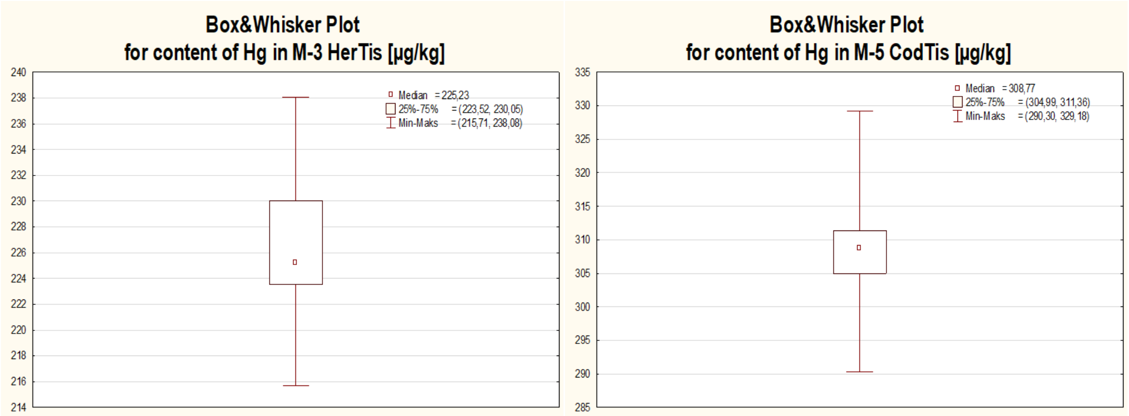

Figure S3. Box&Whisker Plot for the content of Hg in the certified reference material of M-3 Herring Tissue (n = 27) and M-5 Cod Tissue (n = 27) [µg/kg].

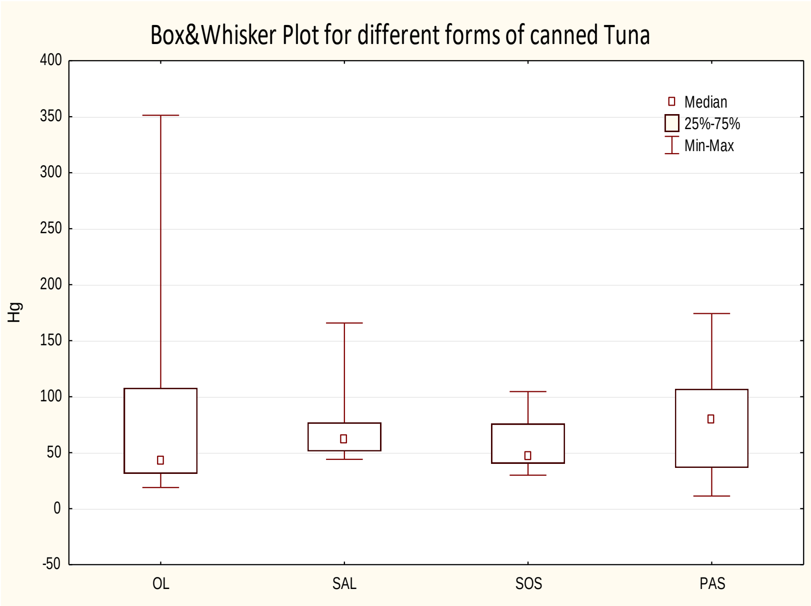

Figure S4. Box & Whisker Plot for content of Hg for canned tuna packed in different matrices (OL- oil, n = 18, median = 43.2; SAL- salads, n = 15, median = 62.6; SOS - own juice and sauces, n = 15, median = 47; PAS – pastes, n = 9, median = 79.2; total n = 57) [µg/kg].

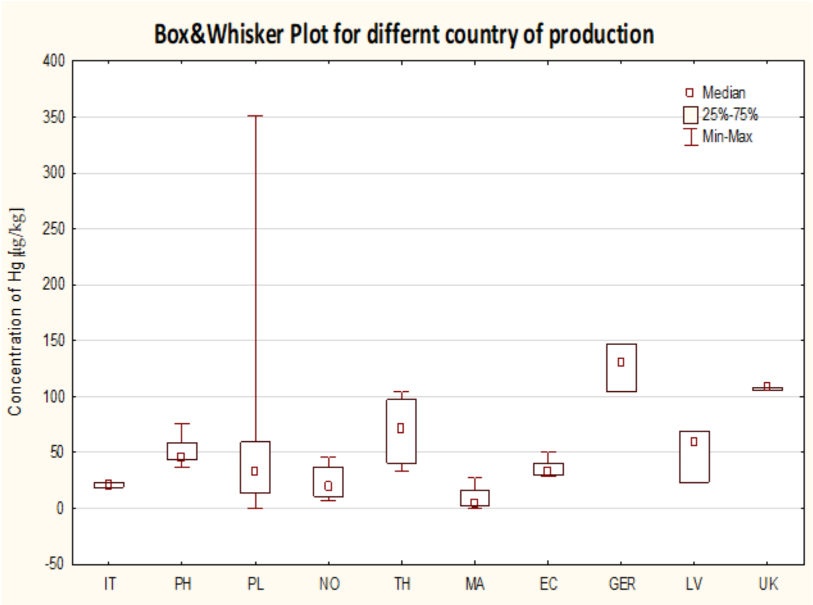

Figure S5. Box & Whisker Plot for content of Hg for canned fish produced in different countries (IT-Italy, n = 3, median = 21.4; PH-Philippines, n = 9, median = 45.6, PL-Poland, n= 192, median = 33.4; NO-Norway, n = 12, median = 19.7; TH-Thailand, n = 9, median = 71.4; MA-Morocco, n = 12; median = 4.6; EC-Ecuador, n = 6; median = 33.2; GER-Germany, n = 3; median = 130; LV-Latvia, n = 3, median = 59.8; UK-United Kingdom, n = 3; median = 107; total n = 252) [µg/kg].

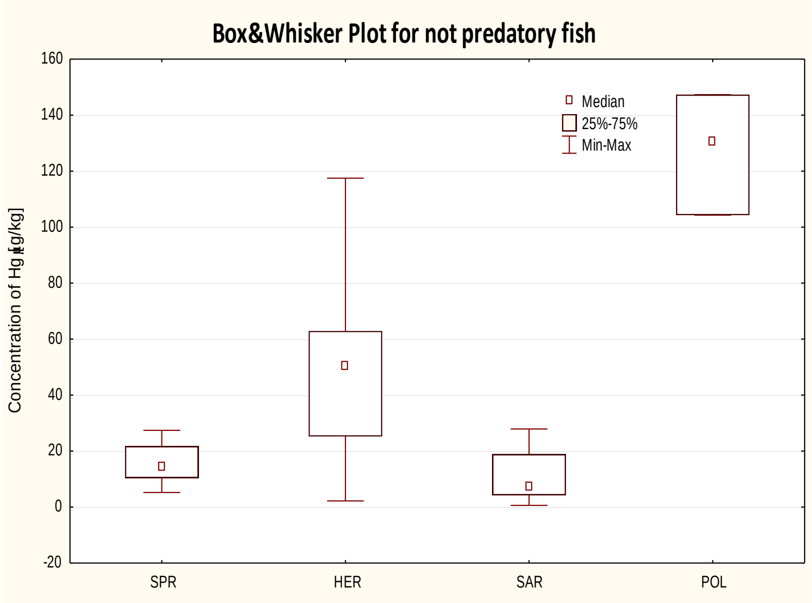

Figure S6. Box & Whisker Plot for content of Hg for not predatory fish (SPR – sprat, n = 27, median = 14.6; HER – herring, n= 54, median = 50.6; SAR – sardine, n = 21, median = 7.3; POL – pollock, n = 3, median = 130; total n = 105) [µg/kg].

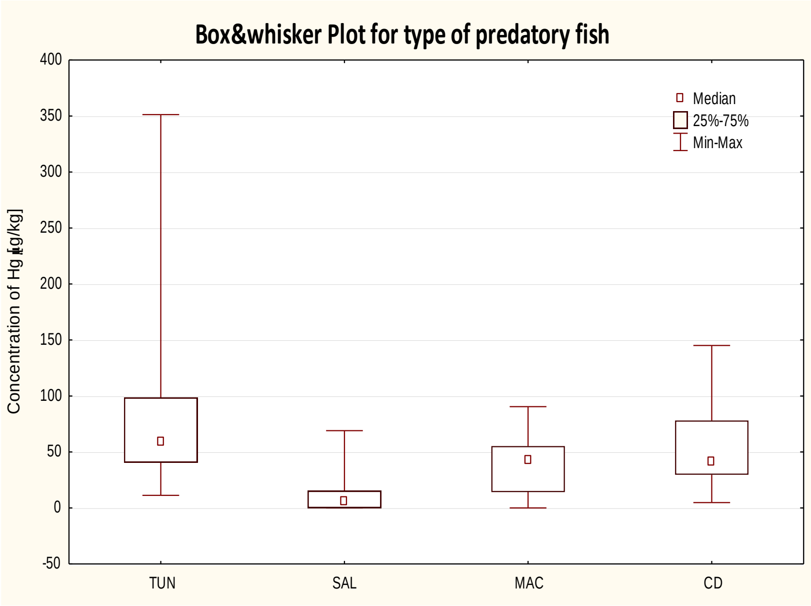

Figure S7. Box & Whisker Plot for content of Hg for type of predatory fish (TUN – tuna, n = 57, median = 58.8; SAL – salmon, n= 24, median = 6.65; MAC – mackerel, n = 42, median = 45.8; CD – cod, n = 24, median = 41; total n = 147) [µg/kg].

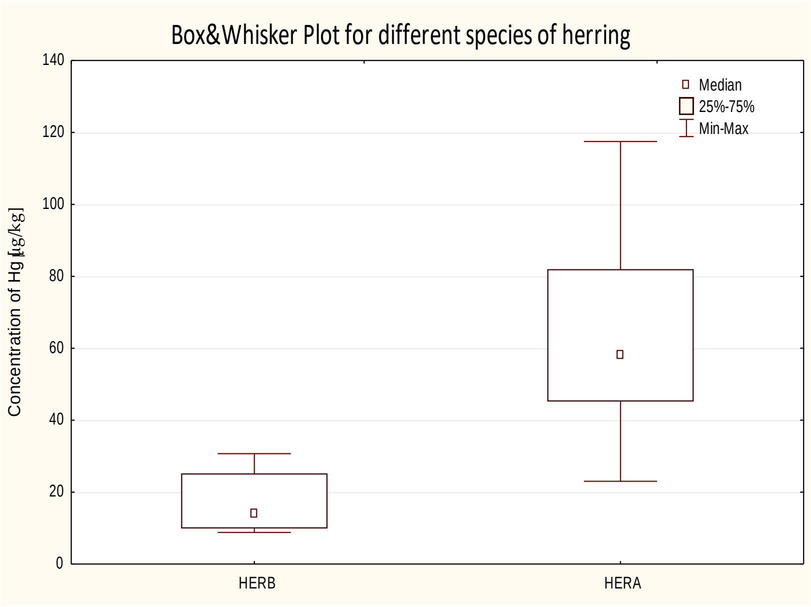

Figure S8. Box & Whisker Plot for content of Hg for species of herring (HERB – Baltic herring, n = 9, median = 13.9; HERA – Atlantic herring, n = 33, median = 58.2; total n = 33) [µg/kg].

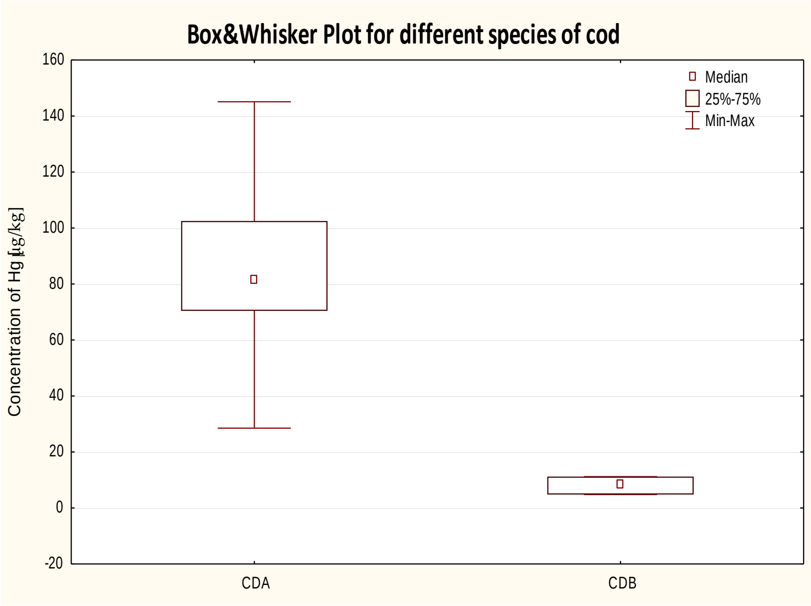

Figure S9. Box & Whisker Plot for content of Hg for two species of cod (CDA – Atlantic cod, n = 9, median = 81.3; CDB – Black cod, n = 3, median = 8.6; total n = 12) [µg/kg].

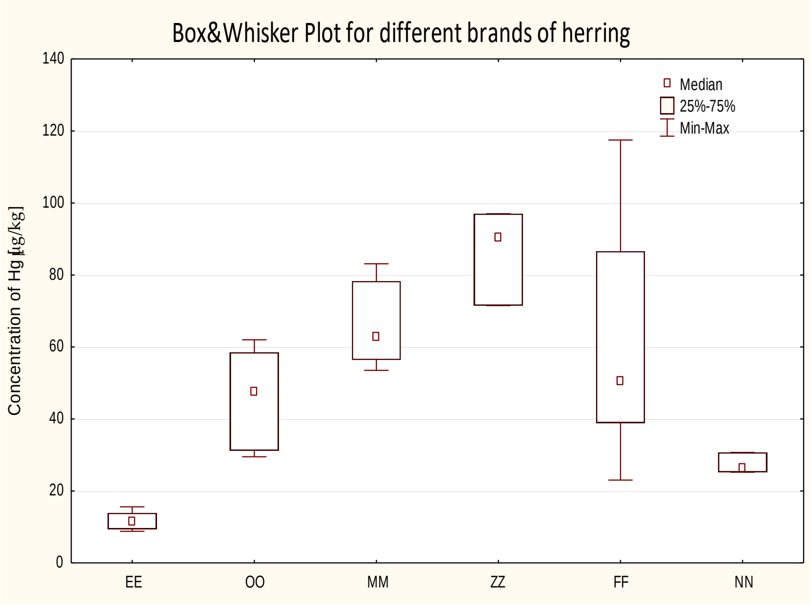

Figure S10. Box & Whisker Plot for content of Hg for considered brands of herring (EE, n = 6, median = 11.3; OO, n = 6, median = 47.4; MM, n = 9, median = 62.9; ZZ, n = 3, median = 90.6; FF, n = 15, median = 50.5; NN, n = 3, median = 26.5; total n = 42) [µg/kg].

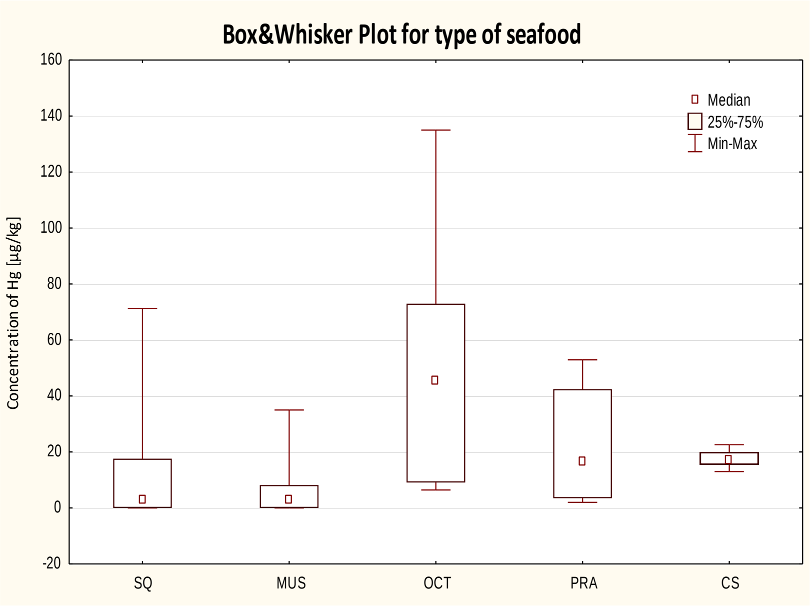

Figure S11. Box & Whisker Plot for content of Hg for type of seafood (SQ – squid, n = 51, median = 2.8; MUS – mussel, n = 18, median = 3.16; OCT – octopus, n = 6,median = 45.5; PRA – prawn, n = 15, median = 16.4; CS – crab sticks, n = 15,median = 17.4; total n = 108) [µg/kg].

References for supplementary materials:

9. Dezfouli, A., Salar-Amolia, J., Ali-Esfahania, T., Hosseinic, H., Ghanatic, K., Evaluating Total Mercury and Methyl Mercury Contents in Canned Tuna Fish of the Persian Gulf, *Iranian Journal of Pharmaceutical Research,* **2018**, 17(2); pp. 585-592.
19. Okyere, H., Voegborolo, R.B., Agorku, S.E., Human expouse to mercury, lead and cadium through consumption of canned mackerel, tuna, pilchard and sardine, *Food chemistry*, **2015**, pp. 331-335.
20. Olmedo, P., Pla, A., Hernández, A.F., Barbier, F., Ayouni, L., Gil, F., Determination of toxic elements (mercury, cadmium, lead, tin andarsenic) in fish and shellfish samples. Risk assessment for the consumers, *Environment International*, **2013**, 59; pp. 63–72.
21. González-Estecha, M., Martínez-García, M.J., Fuentes-Ferrer, M., BodasPinedo, A., Calle-Pascual, A., Ordóñez-Iriarte, J.M., et al., Mercury in canned tuna in Spain. Is light tuna really light? *Food Nutr Sci*, **2013**, 4; pp. 48–54
22. Adil, C., Mustapha, H., Abdeljalil, B., Taoufiq, B., Heavy metals content of canned tuna fish: estimated weekly intake, *Mor. J. Chem*., **2015**, 3(1); pp. 152-156.
23. Dabeka, R. W., Mckenzie, A. D., Forsyth, D. S., Total mercury in canned tuna sold in Canada in 2006, *Food Additives & Contaminants*, 7(2); pp. 110-114. https://doi.org/10.1080/19393210.2013.856036.
24. Andayesh, S., Hadiani, M. R., Mousavi, Z., Shoeibi, S., Lead, cadmium, arsenic and mercury in canned tuna fish marketed in Tehran, Iran, *Food Additives & Contaminants,* **2015**, 8; pp. 93-98, https://doi.org/10.1080/19393210.2014.993430.
25. Vieira, H.C., Bordalo, M.D., Morgado, F., Soares, A.M.V.M., Abreu, S.N., Mercury content in the white and dark muscle of Skipjack tuna (Katsuwonus pelamis) along the canning process: Implications to the consumers, *Journal of Food Composition and Analysis,* **2017**, 56; pp 67-72.
26. Gerstenberger, S.L., Martinson, A., Kramer, J.L., An evaluation of mercury concentrations in three brands of canned tuna, *Environmental Toxicology and Chemistry*, **2009**, 29(2); pp. 237-242. https://doi.org/10.1002/etc.32.
